# Artificial intelligence for the diagnosis and prognosis of leprosy, leishmaniasis, and schistosomiasis: A systematic review

**DOI:** 10.1371/journal.pntd.0014833

**Published:** 2026-11-05

**Authors:** Felipe de Oliveira Franco, Letícia Gabrielle Souza, Claudia Nogueira dos Anjos, Alexandre Dias Porto Chiavegatto Filho

**Affiliations:** Laboratory of Big Data and Predictive Analytics in Health (LABDAPS), School of Public Health, University of São Paulo, São Paulo, Brazil; UFSJ: Universidade Federal de Sao Joao del-Rei, BRAZIL

## Abstract

**Background:**

Neglected Tropical Diseases affect over one billion individuals globally, challenging healthcare systems in endemic regions. While promising, Artificial Intelligence (AI) requires rigorous validation for clinical translation.

**Methodology:**

This systematic review of prediction-model studies evaluated AI applications for leprosy, schistosomiasis, and leishmaniasis (2015–2025). Methodological quality and reporting transparency of the included studies were appraised using the PROBAST+AI and TRIPOD+AI tools, respectively.

**Findings:**

Among 69 studies identified, 89.9% were published after 2020. The literature predominantly focused on diagnostic support (81.2%), leaving the prognosis of adverse outcomes underexplored (18.8%). Methodological fragility was widely observed: 89.9% of models exhibited a high risk of bias, primarily due to lack of external validation (82.6%) and failure to report or appropriately handle missing data (66.7%). Transparency remains a barrier, with source code unavailable in 76.8% of cases, hindering reproducibility.

**Conclusion:**

Current evidence indicates that quantitative output outpaces methodological maturity. Bridging the gap between algorithmic potential and public health impact requires prioritizing open science and robust validation.

## Author summary

Leprosy, leishmaniasis, and schistosomiasis continue to pose significant challenges to global health, disproportionately affecting communities in resource-limited regions. Artificial Intelligence (AI) has emerged as a promising technology to improve how we identify and monitor these diseases. However, the move from laboratory development to clinical practice requires a high level of scientific rigor to ensure that these tools are safe and effective for patient care. By reviewing 69 studies, we found that the recent surge in AI research has not been matched by methodological maturity. Our analysis shows that 89.9% of the developed models were classified as having a high risk of bias. This vulnerability stems from common practices such as using data from only one hospital or failing to test the AI on patients from different geographic areas. Furthermore, scientific transparency remains low, as most researchers do not share the underlying code needed to verify their results. Our study highlights that the current focus on producing more models must be replaced by a commitment to robust validation and open science. For AI to become a reliable tool for clinicians in endemic regions, future research must address these technical shortcomings. Without these changes, AI risks remaining an experimental concept rather than a practical solution for disease control.

## 1. Introduction

Neglected Tropical Diseases (NTDs) represent a group of more than 20 infectious conditions that disproportionately affect populations in socioeconomic vulnerability, especially in low- and middle-income endemic countries [1]. With over one billion individuals affected globally [2], these diseases primarily reach impoverished groups in these regions and are associated with high morbidity and mortality [3–5]. The historical marginalization of NTDs is exacerbated by the low economic return for the pharmaceutical industry, resulting in insufficient investment in research and development. This confluence of human and economic impact positions NTDs as a pressing challenge for global health, imposing a substantial burden on healthcare systems and causing billions of dollars in annual productivity losses [3].

In response to this scenario, global initiatives, such as the World Health Organization (WHO) Road Map for Neglected Tropical Diseases 2021–2030 and target 3.3 of the Sustainable Development Goals (SDGs), have established targets for the control and elimination of these diseases by 2030 [6]. Despite such initiatives, significant gaps in epidemiological surveillance and clinical management still persist [7]. The increasing availability of large-scale clinical and epidemiological data presents a unique opportunity for progress, although the ability to effectively analyze this information to extract actionable insights remains a considerable challenge.

In this context, the application of Artificial Intelligence (AI) algorithms and, primarily, Machine Learning (ML) has emerged as a promising computational paradigm. Unlike traditional statistical approaches, AI models are designed to handle high-dimensional data and identify complex, non-linear relationships among a large number of variables [8]. Given the multifactorial nature of NTDs, which are influenced by a complex interaction of clinical, socioeconomic, demographic, and environmental factors, AI techniques are uniquely positioned to integrate these diverse data streams [9,10]. This capability allows for the development of more accurate diagnostic aid tools and models for the prognosis of adverse outcomes, enabling the optimization of limited resource allocation and the implementation of more targeted and effective public health interventions [11,12].

This systematic review focuses on the application of AI to leprosy, schistosomiasis, and leishmaniasis (visceral and cutaneous). These diseases were strategically selected due to their substantial clinical burden and the diverse public health and clinical management challenges they represent. While leprosy is characterized by persistent diagnostic delays and irreversible physical disabilities, schistosomiasis poses chronic risks of severe systemic complications (such as hepatosplenic disease), and visceral leishmaniasis remains associated with high mortality rates in vulnerable populations. Furthermore, because these conditions are designated as mandatory notifiable diseases in most endemic countries, they are central targets of public health surveillance efforts. Evaluating AI models across this specific subset of diseases provides a comprehensive overview of how machine learning can address key bottlenecks in NTD control, such as predicting disease progression, therapeutic failure, and treatment abandonment under varying health system capacities. Furthermore, the compulsory notification of these conditions in many endemic nations tends to ensure the availability of structured data, which are fundamental for training AI models [13–15]. Thus, the primary objective of this systematic review is to map, synthesize, and critically evaluate the existing literature regarding the use of AI-based prediction models for individual diagnosis and prognosis in human patients affected by these three NTDs. A secondary objective is to explore potential associations between reporting transparency and methodological rigor, aiming to identify research gaps and guide future clinical translation.

While recent literature has explored the growing role of AI tools in NTDs [12,16–18], our review is, to our knowledge, the first to simultaneously apply the Prediction model Risk Of Bias ASsessment Tool + Artificial Intelligence (PROBAST+AI) risk of bias, quality, and applicability assessment tool [19] and the Transparent Reporting of a multivariable prediction model for Individual Prognosis Or Diagnosis + Artificial Intelligence (TRIPOD+AI) reporting guidelines [20]. Specifically, PROBAST+AI evaluates the methodological rigor and potential for biased results, whereas TRIPOD+AI assesses the completeness and transparency of the study’s reporting. Additionally, we investigated the association between scientific transparency and methodological rigor, offering a critical overview aimed at guiding future research and accelerating the clinical translation of these technologies.

## 2. Methodology

### 2.1. Ethics statement

Not applicable. This study is a systematic review of published literature and did not involve primary data collection from human or animal participants.

### 2.2. Protocol, information sources, and eligibility criteria

The protocol for this systematic review was previously registered in the International Prospective Register of Systematic Reviews (PROSPERO), where it is publicly accessible under registration CRD420251137960. The conduct and reporting of the study followed the Preferred Reporting Items for Systematic Reviews and Meta-Analyses (PRISMA) 2020 guidelines and the Transparent Reporting of multivariable prediction models for Individual Prognosis Or Diagnosis tailored for Systematic Reviews and Meta-Analyses (TRIPOD-SRMA) [21] (S1 Table). No protocol deviations occurred during the conduct of this review.

A systematic and comprehensive search was conducted across five electronic databases: IEEE Xplore (Institute of Electrical and Electronics Engineers), ACM Digital Library (Association for Computing Machinery), PubMed, Web of Science, and the Virtual Health Library (Biblioteca Virtual em Saúde - BVS), which aggregates Latin American and Caribbean Health Sciences Literature (LILACS) and the Scientific Electronic Library Online (SciELO). Additionally, studies were included via the Snowball technique, which consisted of screening the reference lists of included articles (backward tracking) and identifying newer papers citing them (forward tracking) to capture relevant literature not retrieved by the primary database search. The full search strings for all databases are provided in S2 Table. The search strategy was designed to maximize sensitivity, combining terms for the diseases of interest with descriptors for predictive models and AI. Primary studies describing the development or validation of AI models for diagnostic aid or prognosis in leprosy, schistosomiasis, and leishmaniasis, published as full articles between January 2015 and July 2025 in English or Portuguese, were included. Studies with non-human populations, in-vitro/in-silico models, reviews, editorials, and abstracts without a corresponding full article were excluded.

### 2.3. Study selection, data extraction, and assessment of bias, quality, applicability, and transparency

The study selection process, outlined in the PRISMA flow diagram (Fig 1), was conducted in two phases by two independent reviewers. Prior to screening, all retrieved records were imported into Zotero reference management software [22] to identify and remove duplicates. Before formal screening and data extraction, a pilot calibration exercise was conducted using a random sample of studies to align interpretations regarding the eligibility criteria, as well as the PROBAST+AI and TRIPOD+AI items. Initially, title and abstract screening was performed, followed by the full-text evaluation of articles that met the initial criteria. Disagreements in both phases were resolved by consensus, with a third reviewer acting as a tie-breaker.

**Fig 1:**
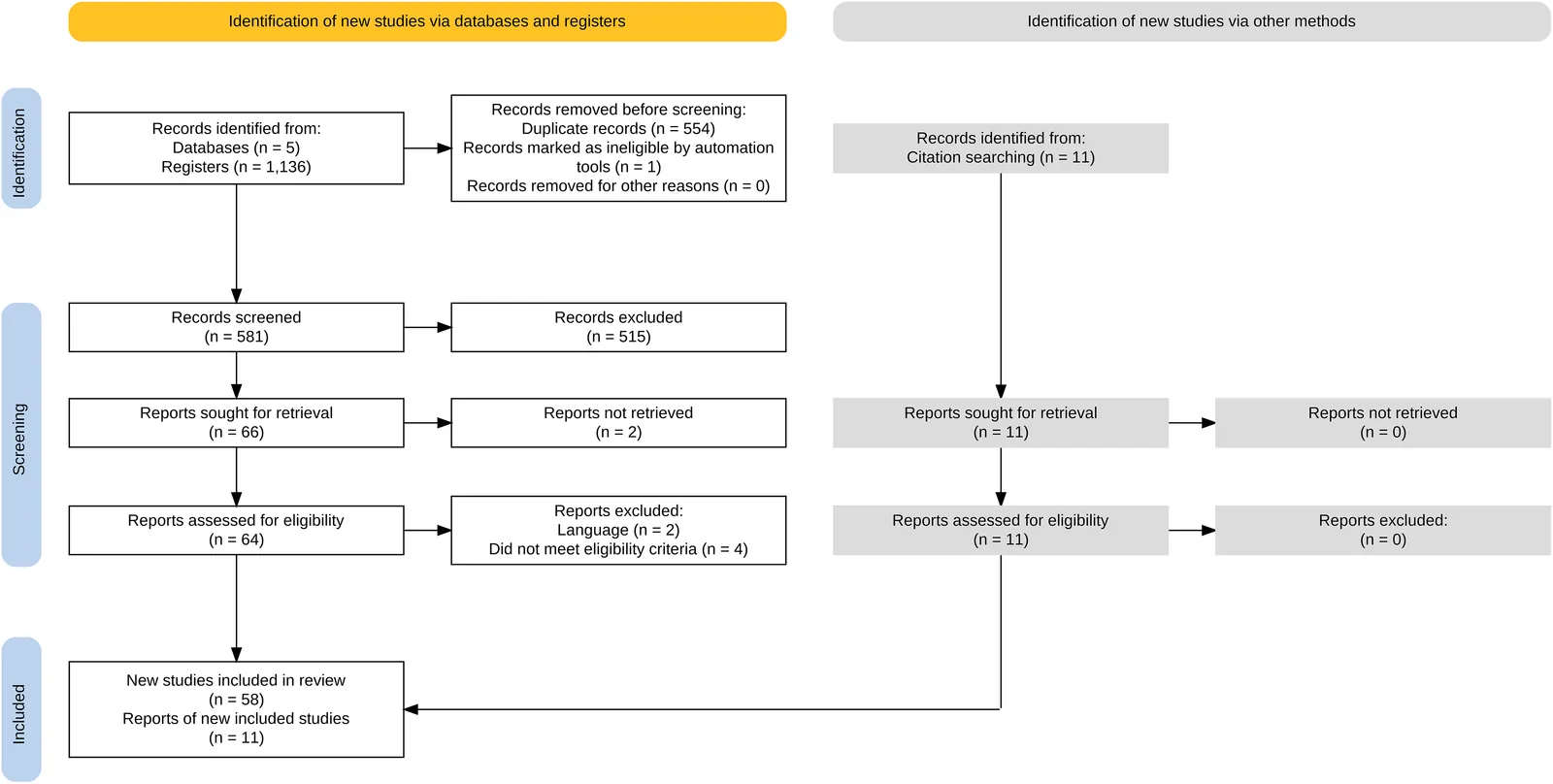
PRISMA flow diagram of the study selection process.

“Diagnostic Aid” was defined as AI tasks for disease detection or species identification in patient samples. “Prognosis and Adverse Outcome Prediction” encompassed models predicting future events, such as disease progression, complications, or mortality.

Data extraction was performed using a standardized form based on the Checklist for Critical Appraisal and Data Extraction for Systematic Reviews of prediction modelling studies (CHARMS) guidelines [23], detailed in S3 Table. A data dictionary was used to define the variables (S4 Table). The risk of bias, quality, and applicability of the models were systematically evaluated using the PROBAST+AI tool [19], which structures the assessment into the domains of participants, predictors, outcome, and analysis (noting that applicability is evaluated only in the first three domains). Following the PROBAST+AI protocol, an overall high risk of bias is assigned if at least one of the evaluated domains scores a high risk. Furthermore, transparency and reporting quality were assessed using the TRIPOD+AI guidelines [20]. TRIPOD+AI, although originally designed to guide manuscript writing, was employed in this review as an external audit instrument. This approach allowed for measuring the transparency and completeness of scientific reporting against current standards of methodological rigor and reproducibility.

### 2.4. Data analysis and statistics

An exploratory data analysis was performed to summarize the main characteristics and plot visualizations. Normality of continuous variables was verified by the Shapiro-Wilk test. Because continuous variables were not normally distributed, non-parametric methods were adopted for bivariate analyses: the Spearman correlation for temporal trends, the Kruskal-Wallis test for comparing sample size medians across target diseases, and Fisher’s Exact Test for categorical associations. To evaluate the association between study characteristics and journal impact factors, Generalized Linear Models (GLMs) were used. Given that impact factor values are strictly positive and right-skewed, a Gamma distribution with a log link was selected, with model adequacy evaluated through deviance and residual inspection.

A significance level of *p* < 0.05 was adopted for all analyses. To account for multiple comparisons, P-values were adjusted using the Benjamini-Hochberg False Discovery Rate (FDR) procedure, computed within four logical hypothesis families: (1) temporal trends in methodological quality and transparency, (2) differences in sample size across target diseases, (3) associations between study characteristics and journal impact factors, and (4) bivariate associations between open science practices and risk of bias or quality concerns.

Statistical computation and graph generation were performed using the Python programming language version 3.12. Data manipulation and statistical tests were executed utilizing the pandas, scipy, and statsmodels libraries, while data visualizations and spatial mapping were created with matplotlib, seaborn, and geopandas. The complete analytical code, including all package dependencies, is publicly available in the study’s GitHub repository.

## 3. Results

### 3.1. Selection flow and general research overview

The study selection process is illustrated in the PRISMA flow diagram (Fig 1). Full bibliographic details for all included studies are provided in S1 Appendix. The initial database search identified 1,136 records. After removing duplicates and applying eligibility criteria in two screening phases, 58 studies were considered eligible and included in the qualitative and quantitative analysis. Specific reasons for excluding articles at the full-text reading stage are listed in S5 Table. To ensure a comprehensive synthesis, the primary search was supplemented by snowball sampling (backward and forward citation tracking), which identified 11 additional relevant studies. Consequently, a total of 69 articles were included in the final qualitative and quantitative synthesis.

The general and publication characteristics of these studies are presented in S6 Table. The analysis of the temporal distribution of publications (Fig 2a) revealed exponential growth in interest in the area. The vast majority of included studies (89.9%) were published in the period from 2020 to 2025, indicating that the application of AI to these NTDs is a rapidly expanding research field. The analysis of funding sources (S6 Table) showed that public funding was predominant (53.6%), but a significant portion of studies (20.3%) did not report their funding source, and 14.5% explicitly stated they received no funding, which may influence the scale and scope of the projects.

**Fig 2:**
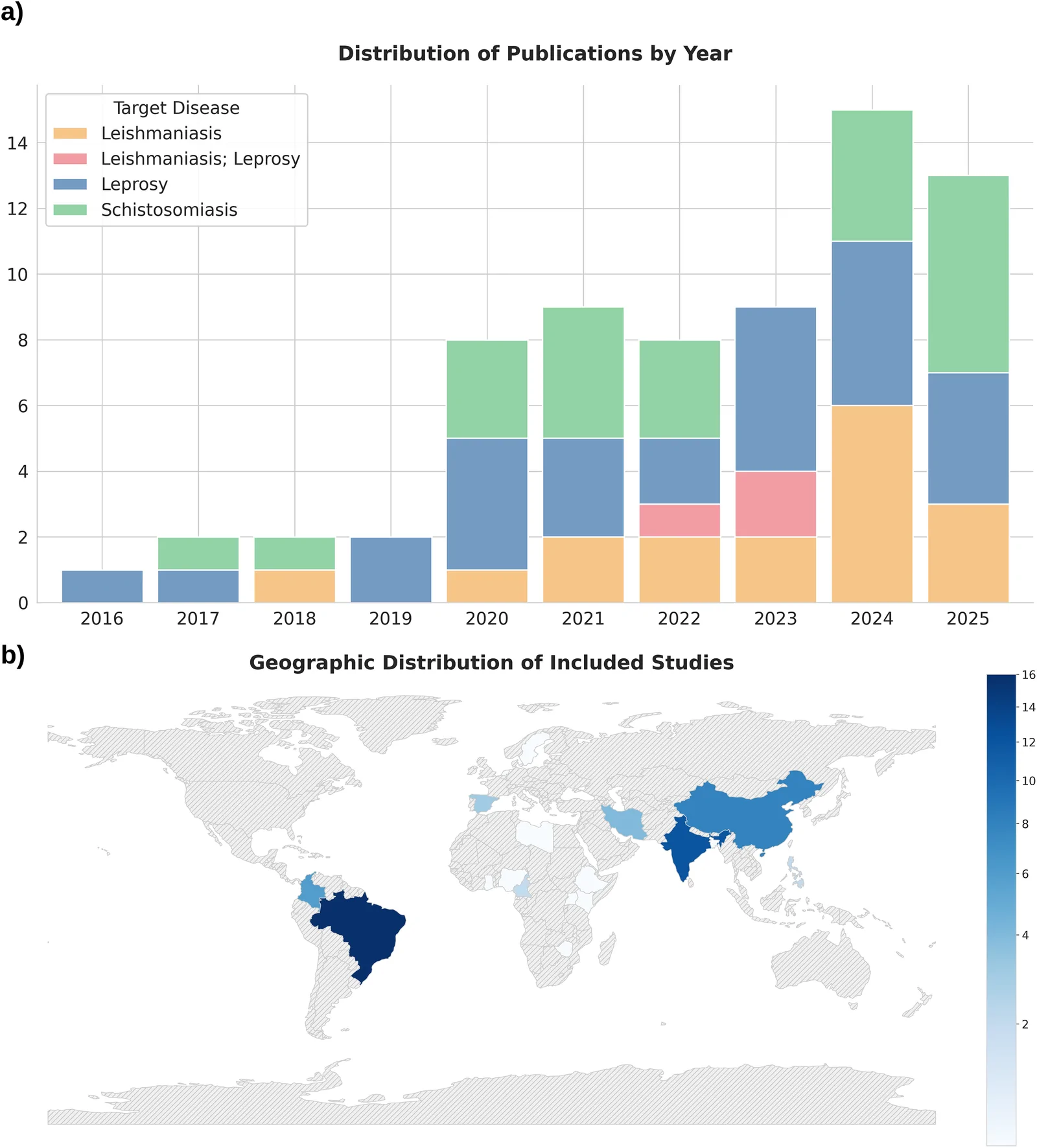
Temporal and geographic distribution of studies. **(a)** Absolute number of publications per year, stratified by disease. **(b)** Choropleth map indicating the global frequency of included studies, highlighting the concentration of research in endemic regions. The base map was generated using the public domain Natural Earth country dataset (available at https://www.naturalearthdata.com/), accessed and plotted via the GeoPandas library in Python.

The geographic distribution of the studies included in the review, visualized in the map in Fig 2b, demonstrates the concentration in countries where NTDs are endemic [1,2]. Brazil led with 21.7% of publications, followed by India (17.4%) and China (11.6%). Together, these three countries accounted for more than half of all scientific production. This pattern suggests that research is strongly driven by local health needs. The analysis by disease (S6 Table) showed that leprosy was the most investigated condition (43.5%), followed by schistosomiasis (31.9%) and leishmaniasis (29.0%). The main prediction task was diagnostic aid (81.2%), reflecting a primary focus on the development of tools for disease identification and classification.

### 3.2. Data characteristics and pre-processing techniques

The characteristics of the data sources, detailed in S7 Table, show a predominance of primary research data (52.2%), single-center studies (50.7%), and proprietary data (55.1%). Statistical analysis (Table 1) revealed a significant difference in sample size between diseases (H = 11.39, p = 0.010), with schistosomiasis studies using substantially larger samples (Median: 1250) compared to leishmaniasis (Median: 172) and leprosy (Median: 300), reflecting the diverse nature of data sources (large microscopy image banks versus smaller clinical cohorts).

**Table 1:** Summary of statistical analyses.

Analysis	Method	Statistic	p (raw)	p (adj)	n
Temporal Trend of RoB	Spearman	Rho = 0.02	0.858	0.858	69
Temporal Trend of Quality Concern	Spearman	Rho = 0.12	0.322	0.430	69
Temporal Trend of Data Availability	Spearman	Rho = 0.36	0.002*	0.008*	69
Temporal Trend of Code Availability	Spearman	Rho = 0.25	0.039*	0.078	69
Difference in Sample Size by Disease	Kruskal-Wallis	H = 11.39	0.010*	0.010*	65
Impact of RoB on IF	GLM (Gamma)	Ratio = 0.97	0.836	0.970	43
Impact of Method. Quality on IF	GLM (Gamma)	Ratio = 1.00	0.970	0.970	43
Data Availability and RoB	Fisher’s Exact	N/A	0.019*	0.075	69
Data Availability and Quality Concern	Fisher’s Exact	N/A	0.069	0.138	69
Code Availability and RoB	Fisher’s Exact	N/A	0.340	0.423	69
Code Availability and Quality Concern	Fisher’s Exact	N/A	0.423	0.423	69

N: Number of studies included in the analysis.

Spearman: Spearman correlation coefficient (Rho).

Kruskal-Wallis: H test statistic. Compares medians among multiple groups.

Fisher’s Exact: Test of association for categorical variables.

GLM (Ratio): Generalized Linear Model (Gamma family, Log link), reporting the exponentiated coefficient ($e^{\beta }$). Impact Factor showed deviation from normality (Shapiro-Wilk W = 0.691, p-value < 0.001), with model fit verified by residual diagnostics (deviance to degrees of freedom ratio = 0.315).

* indicates a p-value < 0.05.

Abbreviations: RoB, Risk of Bias; IF, Impact Factor; N/A, Not Applicable; p (raw): unadjusted p-value; p (adj): False Discovery Rate (FDR) adjusted p-value using the Benjamini-Hochberg procedure, controlled within hypothesis families.

The unit of analysis varied, with a significant portion (62.3%) using images (macroscopic or microscopic) as the primary input. This focus on visual data is corroborated by the analysis of predictor categories (S7 Table), where “image” was the most frequent (66.7%). The word cloud in S1 Fig reinforces this observation, illustrating the dominance of image variables while also highlighting the frequent inclusion of demographic and clinical features.

Data pre-processing techniques, summarized in S8 Table, revealed critical methodological gaps and a pattern of widespread underreporting. The management of missing data was not reported in 66.7% of the studies, which compromises the robustness of the models. Similarly, the management of class imbalance, a common problem in health data, was not reported in 50.7% of cases. While some form of modification to continuous predictors was applied in 27.5% of cases, the management of categorical predictors was even less reported, with only 4.3% of studies explicitly describing the use of techniques for this purpose.

### 3.3. Model architecture, training, and validation

The studies explored a wide range of AI algorithms, with more than 200 mentions of different techniques tested, as detailed in S9 Table. S2a Fig illustrates the 10 most frequently tested algorithms, with a focus on classic models such as Random Forest, Support Vector Machine, and Logistic Regression. A substantial portion of the studies (37.6%) used transfer learning (S9 Table). It is notable that the application of this technique was exclusively in studies that analyzed images. No study based on tabular data (i.e., structured clinical, demographic, or laboratory features presented in a matrix format) reported the use of transfer learning, indicating that its potential to leverage knowledge from rich data domains for scenarios with sparser data remains unexplored. Hyperparameter optimization, a critical determinant for model performance, was reported in a structured manner (automated or defined searches) in only 53.6% of the studies, while 15.9% did not report the process and 23.2% relied on manual adjustment, which is less reproducible. Additionally, the analysis of the data split strategy (S8 Table) revealed that although the use of a simple train/test split was the most common (42.0%), a considerable portion used methods such as cross-validation (21.7%) or a train/validation/test split (23.2%). However, 8.7% of the studies did not report their data splitting strategy.

The Sankey diagram in Fig 3 provides an integrated view of the model development flow. It illustrates frequently observed methodological paths, such as flows connecting leishmaniasis-related research to diagnostic aid tasks, predominantly culminating in Deep Learning models.

**Fig 3:**
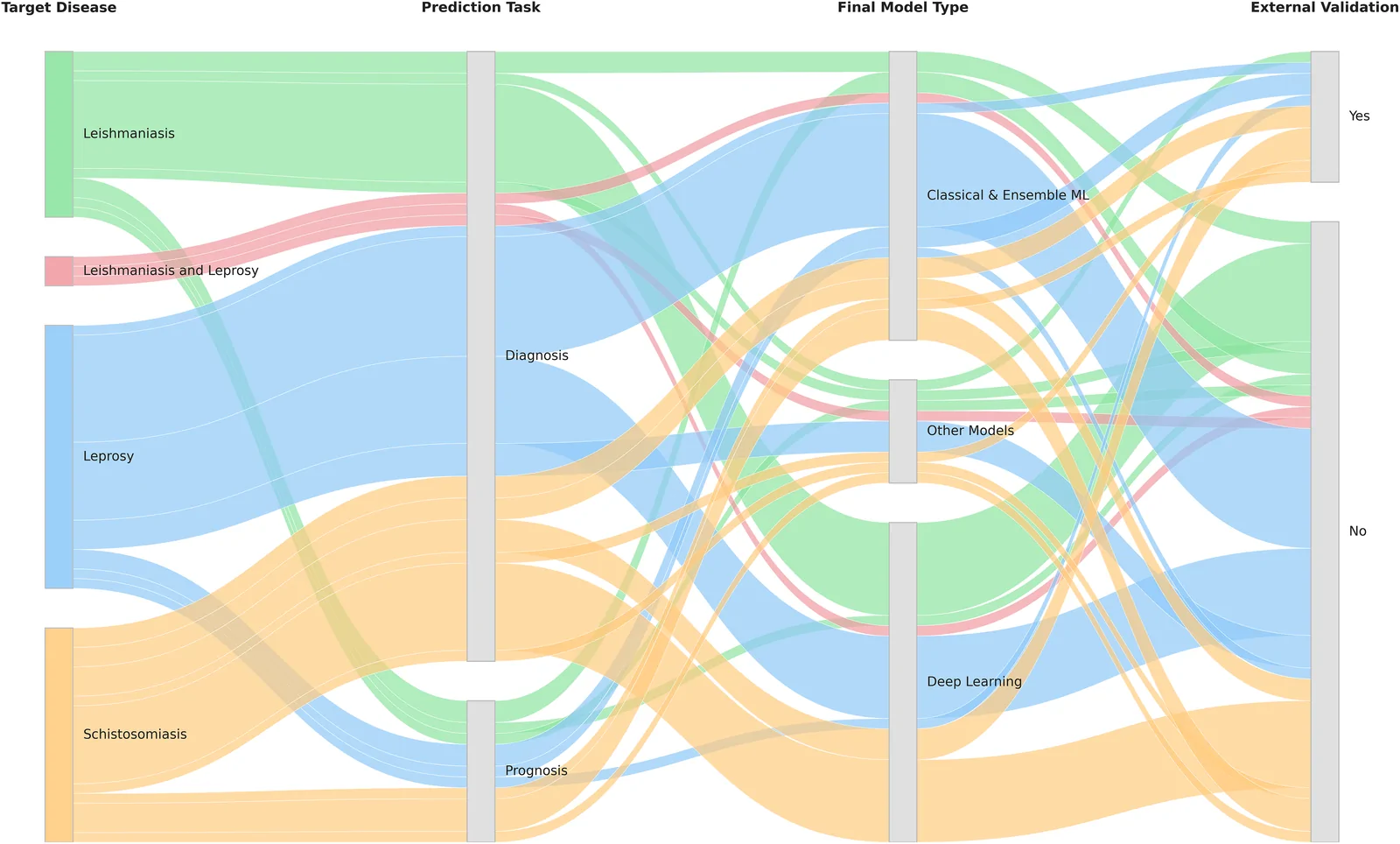
Methodological flow of model development. Sankey diagram illustrating the associations between key study characteristics. The flow progresses from left to right, starting with the Target Disease, followed by the Prediction Task (Diagnosis or Prognosis), the Final Model Type, and concluding with the External Validation status (Yes or No). Colors represent the Target Disease: Green corresponds to Leishmaniasis, Blue to Leprosy, Orange to Schistosomiasis, and Pink to studies addressing both Leishmaniasis and Leprosy. The width of the connections is proportional to the number of studies sharing these specific characteristics. The variable “Final Model Type” is derived from an aggregation of the variable “Final Model”. The width of the connections is proportional to the number of studies sharing these characteristics.

In contrast, an equally strong flow shows leprosy studies, also focused on diagnostic aid, but resulting predominantly in Classical & Ensemble models. However, the most critical and unifying observation derived from Fig 3 is the final stage of the process: regardless of the disease, task, or model type, the vast majority of research flows converge on the absence of external validation. In this context, 82.6% of the studies did not validate their models on an independent dataset (S9 Table). A detailed comparison of study characteristics, data modalities, model architectures, and validation practices stratified by target disease is provided in S10 Table.

### 3.4. Reported performance, transparency, and reproducibility

As summarized in S11 Table, studies tended to report high predictive performance, with a mean Area Under the Receiver Operating Characteristic Curve (AUC ROC) of 0.86 (median 0.86, Interquartile Range [IQR] 0.80-0.94) and a mean F1-Score of 0.90 (median 0.93, IQR 0.84-0.98). However, these values should be interpreted with caution, as AUC ROC was not reported in 65.2% of the studies. This reporting gap suggests that the pooled performance metrics represent a specific subset of the literature, potentially reflecting an overestimation of results. Furthermore, these metrics are not directly comparable due to substantial heterogeneity in data sources, prediction tasks, validation strategies, and outcome definitions.

Transparency and reproducibility, analyzed in S12 Table and S3 Fig, emerged as key challenges for the field. Analysis revealed a significant gap in research artifacts: source code was not reported or made available in 76.8% of studies, while access to data was absent in 42.0% of cases. This closed science paradigm significantly obstructs independent verification, replication of findings, and iterative model development by the scientific community.

Furthermore, model interpretability remained a marginal consideration, with over half of the studies (52.2%) failing to employ any Explainable Artificial Intelligence (XAI) technique. These techniques describe how algorithms arrive at their predictions. Among the studies that did address this aspect, 23.2% utilized inherently interpretable models, such as decision trees and logistic regression, while post-hoc explanation techniques, such as SHapley Additive exPlanations (SHAP) (4.3%) and Gradient-weighted Class Activation Mapping (Grad-CAM) (7.2%), were less frequent. Specifically, SHAP provides a model-agnostic approach to quantify how individual predictors contribute to a prediction, whereas Grad-CAM is applied to image-based models to generate heatmaps highlighting which regions of the original image most influenced the model’s decision. Most studies (69.6%) evaluated their performance solely against another AI model, while a minority (7.2%) used a clinical gold standard as a baseline, limiting the assessment of practical utility. This gap is further reflected in clinical translation metrics: only 27.5% of the studies proposed a concrete clinical tool. Instead, the majority remained confined to preliminary proofs of concept (33.3%) or interpretability analyses (27.5%), indicating that the field is still in an exploratory stage and far from clinical implementation.

### 3.5. PROBAST+AI and TRIPOD+AI

The methodological quality assessment performed using the PROBAST+AI tool demonstrated high quality concerns in 85.5% of the studies during the model development phase, while 89.9% were classified as having an overall high risk of bias during the model evaluation phase (Fig 4). These results were primarily conditioned by failures in the analysis domain, which concentrates deficiencies in the management of missing data, potential overfitting problems, and the lack of external validation.

**Fig 4:**
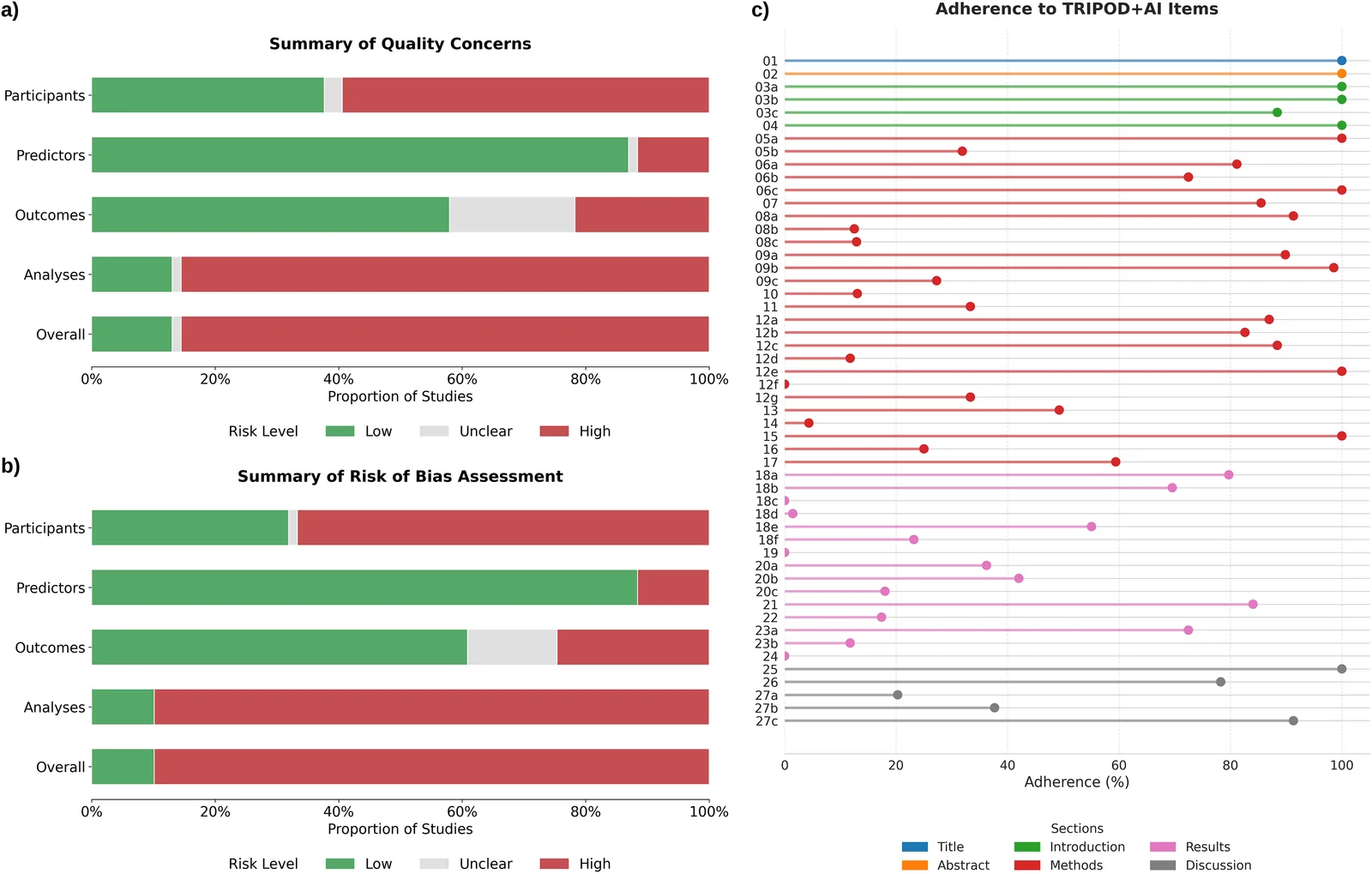
Assessment of PROBAST+AI and TRIPOD+AI. **(a)** Distribution of quality concerns across PROBAST+AI domains for the model development phase. **(b)** Distribution of risk of bias across PROBAST+AI domains for the model evaluation phase. **(c)** Adherence rate (% of studies) to individual items of the TRIPOD+AI checklist, grouped by the structural sections of the scientific article.

In PROBAST+AI, applicability refers to concerns that the participant population, predictor definitions or measurements, or outcome definitions and timing may not align with the reviewer’s specific clinical question or intended use of the model. Concern regarding clinical applicability was low in 81.2% of cases for model development and 82.6% for model evaluation (S4 Fig), indicating that the predictors and outcomes reported were generally commensurate with the review’s scope.

Adherence to the TRIPOD+AI reporting guidelines showed significant variations across article sections (Fig 4c). A detailed item-by-item breakdown of adherence rates across the 69 studies is provided in S13 Table. Basic identification and most introduction items adhered in their totality to reporting standards, while specific methodological elements showed lower compliance. Notably, only 33.3% of the studies detailed missing data management, and even fewer provided a justification for the sample size (13.0%) or addressed algorithmic fairness (4.3%). This variability in reporting hinders the assessment of internal validity and restricts experimental reproducibility.

Statistical analyses confirmed associations between transparency practices and methodological rigor (Table 1). Temporal trend analyses revealed no significant improvement in the overall risk of bias (Rho = 0.02; FDR p = 0.858) or quality concerns (Rho = 0.12; FDR p = 0.430) over the years, quantitatively confirming that methodological rigor has remained stagnant despite the recent surge in publications. A significant positive correlation was observed between the year of publication and the availability of data (Rho = 0.36; FDR p = 0.008). Code availability demonstrated a similar positive, though marginal, trend over time (Rho = 0.25; FDR p = 0.078). Following multiple testing correction, the availability of raw data indicated a borderline association with a lower risk of bias (FDR p = 0.075). However, this association was not observed for code availability (FDR p = 0.423), indicating that opening the algorithm does not, in isolation, ensure the quality of methodological development. Finally, no significant association was detected between the journal impact factor and the risk of bias (FDR p = 0.970) or quality concern (FDR p = 0.970).

## 4. Discussion

### 4.1. Research overview, quality, and methodological challenges

This systematic review of 69 studies on the application of AI to leprosy, schistosomiasis, and leishmaniasis reveals a research field characterized by a disconnect between its rapid expansion and its methodological maturity. Our results, consolidated through a comprehensive assessment using the PROBAST+AI [19] and TRIPOD+AI [20] guidelines, point to a scenario of conflicting trends. The evidence suggests that while the volume of publications has surged exponentially since 2020, this quantitative advance has not been accompanied by a corresponding qualitative improvement. Similar patterns have been reported in systematic reviews of machine learning–based clinical prediction models, which consistently describe high publication growth alongside persistent risks of bias and limited use of external validation [24,25].

Assessments using the PROBAST+AI tool revealed a high risk of bias in the vast majority of studies, a finding that calls into question the validity of most published results and suggests that the field, despite its vitality, operates on a fragile methodological base. This fragility is particularly concerning when considering the potential of AI in diagnostic aid and the prediction of adverse outcomes in vulnerable populations. The use of models with a high risk of bias in these NTDs contexts can result in “digital neglect”. Instead of mitigating inequalities, poorly validated clinical decision support tools risk reproducing technical inaccuracies and perpetuating healthcare disparities [26], making it difficult to correctly stratify risk and efficiently allocate resources in already challenging scenarios.

The widespread underreporting of crucial methodological steps, such as missing data management, class imbalance handling, and data splitting strategies, undermines the credibility of the findings. This fragility is deepened by the predominance of simple train/test splits, which, although common, are more susceptible to optimistic results and are less robust. More critically, the almost complete absence of external validation suggests that the high reported performances, such as the mean AUC ROC of 0.86, are likely overestimated and reflect statistical optimism rather than generalizable robustness [25,27]. These systematic omissions in reporting crucial methodological steps, therefore, not only prevent reproducibility but also sustain the high risk of bias identified, suggesting that the field may be developing models with overestimated performance and little robustness in real-world scenarios. Recent disease-specific reviews strongly corroborate this assessment. In the context of leprosy, Fernandes et al. [28] identified a critical deficit in external validation and poor adherence to open science practices, particularly regarding code and data availability. Similarly, Yohannes et al. [29] observed that diagnostic models for skin NTDs frequently carry a high risk of bias driven by data scarcity, reliance on small private cohorts, and inadequate multimodal alignment.

It is important to acknowledge, however, that the maturity of AI research in NTDs is evolving. Earlier studies often represented preliminary proofs-of-concept, where single-center designs and the lack of external validation were expected and methodologically appropriate to establish initial feasibility and cost-effectiveness. Nevertheless, as the field progresses, the persistent lack of robust validation in more recent publications remains a barrier to large-scale implementation.

We identified a positive temporal trend toward open science, with more recent studies being more likely to share data and code. However, this advance in transparency did not coincide with an improvement in overall methodological rigor, which remained stagnant. Our analysis offers an important nuance: while conservative multiple testing corrections attenuated strict statistical significance, data availability still demonstrated a marginal association with lower risk of bias (FDR p = 0.075). This finding does not establish causality but raises the hypothesis that the practice of preparing a dataset for public scrutiny may be a marker of a more diligent and methodologically rigorous research approach from its conception. In this sense, the unavailability of source code in 76.8% of cases represents a critical barrier to scientific reproducibility. In a global health scenario, transparency is not just an academic ideal but a safety requirement. The lack of access to algorithms prevents research centers in endemic countries from auditing, adapting, and validating these tools in their own epidemiological realities, maintaining dependence on technologies whose internal mechanisms remain inaccessible for independent verification.

The field exhibits great diversity in the exploration of algorithms but a notable convergence on simpler or well-established final models. The application of transfer learning techniques was exclusively in image-based studies. This is a notable gap, as the potential of the technique to accelerate model development in tabular data, which are abundant in healthcare systems, remains underexplored [30,31]. This distinction between image-based and tabular data studies is also reflected in technical choices. The analysis of algorithms (S2 Fig) reveals a clear technical bifurcation: a preference for models such as Random Forest and Support Vector Machine for structured data, and the prominence of deep learning architectures, such as YOLOV5, for image tasks.

Beyond this technical division, managing these three diseases involves distinct biological and clinical challenges. Leprosy is caused by Mycobacterium leprae, whereas schistosomiasis and leishmaniasis are parasitic infections. Diagnostically, finding parasite eggs in a noisy stool smear demands different computer vision strategies compared to identifying bacterial bacilli in human tissue. Similarly, prognostic models must navigate entirely different clinical trajectories, such as predicting irreversible nerve damage in leprosy versus severe hepatosplenic complications in schistosomiasis. Future research should explicitly account for these pathogen-specific characteristics and clinical pathways.

The scarce use of XAI techniques represents a barrier to trust and implementation. Most studies did not employ any interpretability method, which limits clinical validation, bias detection, and trust-building with healthcare professionals [32,33]. In the management of NTDs, where diagnostic aid and the prediction of complications often occur in resource-limited settings, the “black-box” nature of the models can be a technical and cultural obstacle. Without tools that allow the healthcare professional to understand the prediction criteria, trust in the technology may be compromised. This scenario makes it difficult to accept AI as a clinical decision support and health surveillance tool that is safe, auditable, and reliable for monitoring the clinical evolution and adverse outcomes of patients.

Our results confirm the predominant focus of the literature on diagnostic aid tasks. However, our mapping also reveals an incipient, though growing, exploration of prognostic and risk stratification applications. Thus, this review highlights the emerging role of AI beyond simple disease identification, encompassing the prediction of clinical evolution, severity, sequelae, and mortality.

### 4.2. Contextualization in the literature and implications for public health

The lack of external validation, observed in 82.6% of the studies, constitutes a significant barrier to the broad scalability of AI solutions in NTDs. While external validation is not always a strictly mandatory prerequisite for every application, particularly when models offer immediate value for addressing specific challenges within a local context, it remains a primary goal for clinical translation. Nonetheless, the absence of testing on independent datasets limits the understanding of the transportability of these tools. The fundamental risk lies in the generalization of conclusions and the attempt to expand these models to new geographic areas without proper proof of robustness, which can lead to unexpected drops in performance in different populations.

This lack of external validation, combined with methodological underreporting, represents a recurring challenge for the clinical translation of AI models in various fields of medicine [18]. Our observation that most studies are based on single-center (50.7%) and proprietary (55.1%) data reinforces this concern, as dependence on local cohorts is a known barrier to the transportability of models to other clinical or demographic contexts. This dependence on restricted datasets is a broader bottleneck in tropical medicine. Attai et al. [34] noted that the performance of these models is heavily constrained by small sample sizes and the lack of publicly available training data in resource-poor settings. In the specific case of leishmaniasis, Sadeghi et al. [35] emphasizes that existing datasets often originate from older timeframes and fail to capture contemporary shifts in disease manifestation, limiting the reliability of the models. The WHO Global report on neglected tropical diseases 2025 highlights that persistent data gaps and weak surveillance systems undermine strategic planning [2]. This is a macro statement that aligns with the methodological weaknesses identified in this review regarding data quality and the lack of robust pre-processing practices.

However, beyond a general overview of weaknesses, the analysis of exemplary studies within our own review offers a concrete roadmap for the future of the field. These works demonstrate that it is possible to combine technical innovation with best practices of rigor and transparency. For example, while our analysis pointed to the rare use of external validation, the work of Tió-Coma et al. (2021) [36] serves as a methodological gold standard by developing a prognostic signature for leprosy using a prospective design with independent discovery and validation sets. The transparency of the study, with the deposition of raw data in a public repository such as the NCBI Gene Expression Omnibus (GEO), solidifies its status as a model for the development of clinically relevant tools. Similarly, responding to the lack of generalization and transparency we identified, Ward et al. (2022) [37] represents a model of open science by developing a diagnostic system for helminths validated in four distinct endemic countries and by making its image dataset and code available on the Kaggle platform.

Finally, while most studies focus on simple classification tasks, more recent works such as those by Zhi et al. (2025) [38] and Sadeghi et al. (2024) [39] point to the next methodological frontier. The former advances toward complex modeling of multimorbidity in schistosomiasis using Graph Neural Networks, and the latter addresses the “black-box” critique by applying XAI techniques to make leishmaniasis diagnostic aid models more transparent and auditable. Together, these studies are a guide for future research can translate the potential of AI into real impact for public health in a transparent and reproducible way.

The predominance of studies focused on diagnostic aid (81.2%), especially using images, is understandable given the visual nature of many cutaneous NTDs manifestations, such as in leishmaniasis and leprosy [16]. While practical applications of these models are underway, including the WHO Skin NTDs mobile application, their clinical effectiveness remains dependent on rigorous real-world validation [40]. However, the scarcity of prognostic models (18.8%) represents an underexplored strategic opportunity. Prognostic models capable of predicting critical outcomes (such as the development of disabilities in leprosy or progression to hepatosplenic forms in schistosomiasis) would have a transformative impact on both individual clinical management and public health resource allocation. The lack of focus on prognosis limits the potential of AI to move from an identification tool to a tool for managing and preventing adverse outcomes.

For an AI model to translate into a clinical decision support tool, it is essential that its added value be demonstrated beyond predictive accuracy, either through integration into the clinical workflow or through measurable benefits in cost, speed, or accessibility, as recommended by recent evaluation guidelines [41,42]. However, the clinical utility of a model remains uncertain not only when comparisons with existing standards of care are lacking but also when its reported performance is derived from small sample sizes and without proper external validation, as observed in most of the analyzed studies.

### 4.3. Study limitations

This review has some limitations that should be considered. First, although our search strategy was comprehensive and systematic, the inclusion of studies only in English and Portuguese may have led to the exclusion of relevant studies published in other languages, particularly Spanish, as it is the primary language in several endemic regions. Second, while the absence of the Embase database constitutes a limitation, this impact was mitigated by employing a search strategy that integrated multiple global and regional databases, supplemented by snowball sampling, to ensure broad coverage of literature from NTD-endemic areas. Additionally, indexing lag and publication bias may have excluded some recent studies. Third, methodological underreporting itself, which is one of the main findings of this study, imposes a limitation on our analysis. The lack of reporting on missing data management, for example, prevents an in-depth assessment of the imputation versus exclusion techniques used in practice, forcing us to analyze the report itself rather than the underlying practice. Finally, our statistical analysis of the field dynamics is exploratory in nature. The associations found, although statistically significant, do not imply causality and should be interpreted as hypotheses for future scientific investigations. Particularly, the Generalized Linear Model (GLM) was not adjusted for covariables due to sample size constraints, which mitigated the risk of model overfitting. Furthermore, the included studies exhibit heterogeneity across target diseases, prediction tasks, outcome definitions, and data modalities. This methodological diversity limits the feasibility of robust cross-study comparisons and quantitative meta-analysis, thereby restricting the broad generalizability of inferential findings.

## 5. Conclusion

The application of AI to NTDs is a rapidly evolving field, but one that currently suffers from systemic methodological weaknesses that threaten its clinical relevance. Future progress will depend not only on the development of more sophisticated algorithms but, more importantly, on the adoption of a culture of rigor, reproducibility, and transparency. Based on our findings, several directions for future research can strengthen the field. Greater adherence to reporting guidelines, such as TRIPOD+AI, could mitigate underreporting and improve the overall quality of studies. The continuity of open science practices, especially the sharing of curated data, also seems to be a promising path to increase methodological rigor. Strategically, expanding the research focus beyond diagnostic aid, exploring prognostic models, and including robust comparisons with existing standards of care is crucial to demonstrate the added value of new technologies. Finally, there is vast technical potential to be explored, including the application of transfer learning for tabular data [30,31] and the routine incorporation of XAI techniques [32,33] to transform models into more transparent and auditable tools.

By addressing these methodological and strategic gaps, the research community can ensure that the potential of AI is effectively translated into robust, reliable, and equitable tools. Only then will we be able to make a real difference in the lives of the world’s most vulnerable populations, in alignment with the goals of the WHO Road Map for 2030. Furthermore, considering the rapid expansion of the field, updating this review within the next three to five years would be useful to assess whether methodological robustness is keeping pace with the growing volume of literature.

## Supporting information

S1 Table TRIPOD-SRMA checklist for reporting systematic reviews of prediction model studies. This checklist is sourced from: Snell KIE, Levis B, Damen JAA, et al. Transparent reporting of multivariable prediction models for individual prognosis or diagnosis: checklist for systematic reviews and meta-analyses (TRIPOD-SRMA). BMJ. 2023;381:e073538. Note: N/A - Not applicable. Not applicable as no meta-analysis was conducted.
(PDF)

S2 Table Full search strategy for each database. Abbreviations: ACM, Association for Computing Machinery; BVS, Biblioteca Virtual em Saúde (Virtual Health Library); IEEE, Institute of Electrical and Electronics Engineers; LILACS, Latin American and Caribbean Health Sciences Literature; MeSH, Medical Subject Headings; SciELO, Scientific Electronic Library Online.
(PDF)

S3 Table Data extraction framework based on CHARMS domains. Abbreviations: AUC-ROC, Area Under the Receiver Operating Characteristic curve; CHARMS, Checklist for Critical Appraisal and Data Extraction for Systematic Reviews of Prediction Modelling Studies; ID, Identifier; LOOCV, Leave-One-Out Cross-Validation; ML, Machine Learning; XAI, Explainable Artificial Intelligence.
(PDF)

S4 Table Data Dictionary. Abbreviations: AUC-ROC, Area Under the Receiver Operating Characteristic curve; ID, Identifier; LOOCV, Leave-One-Out Cross-Validation; ML, Machine Learning; XAI, Explainable Artificial Intelligence.
(PDF)

S5 Table Reasons for exclusion of studies after full-text screening. References regarding the excluded studies are presented in S2 Appendix.
(PDF)

S6 Table General cCharacteristics. Abbreviations: SD, Standard Deviation; IQR, Interquartile Range; Min, Minimum; Max, Maximum. Note: The category “Diagnosis” in this table corresponds to “Diagnostic Aid” as defined in the methodology, encompassing disease detection and species identification. The category “Prognosis” refers to “Prognosis of Adverse Outcomes”, including the prediction of disease progression, complications, and mortality. Disease staging was classified according to its role in predicting clinical evolution versus current status confirmation.
(PDF)

S7 Table Data characteristics. * The sum of frequencies and percentages may exceed the total number of studies (69) and 100%, as a single study may contain multiple values for the same characteristic. The values reflect the count of each individual mention. Abbreviations: SD, Standard Deviation; IQR, Interquartile Range; Min, Minimum; Max, Maximum.
(PDF)

S8 Table Data pre-processing characteristics. * The sum of frequencies and percentages may exceed the total number of studies (69) and 100%, as a single study may contain multiple values for the same characteristic. The values reflect the count of each individual mention. Abbreviation: LOOCV, Leave-One-Out Cross-Validation.
(PDF)

S9 Table Machine learning model details. * The sum of frequencies and percentages may exceed the total number of studies (69) and 100%, as a single study may contain multiple values for the same characteristic. The values reflect the count of each individual mention. Abbreviations: ML, Machine Learning; XGBoost, eXtreme Gradient Boosting.
(PDF)

S10 Table Study characteristics and methodological indicators stratified by target disease. Note: IQR, Interquartile Range; ML, Machine Learning. Data and source code availability include resources designated as publicly accessible or available upon request.
(PDF)

S11 Table Reported model performance. Abbreviations: SD, Standard Deviation; IQR, Interquartile Range; AUC ROC, Area Under the Receiver Operating Characteristic Curve; Min, Minimum; Max, Maximum. Note: The mean and median values reported reflect only the subset of studies that disclosed each specific metric. The high percentage of “Not available” data indicates a significant reporting bias in the literature.
(PDF)

S12 Table Transparency, interpretation, and clinical utility. * The sum of frequencies and percentages may exceed the total number of studies (69) and 100%, as a single study may contain multiple values for the same characteristic. The values reflect the count of each individual mention. Abbreviations: XAI, Explainable Artificial Intelligence; Grad-CAM, Gradient-weighted Class Activation Mapping; SHAP, SHapley Additive exPlanations; URL, Uniform Resource Locator.
(PDF)

S13 Table TRIPOD+AI Checklist. Footnotes: D - items relevant only to the development of a prediction model; E - items relating solely to the evaluation of a prediction model; D;E - items applicable to both the development and evaluation of a prediction model. This checklist is adapted from: Collins GS, Moons KGM, Dhiman P, et al. TRIPOD+AI statement: updated guidance for reporting clinical prediction models that use regression or machine learning methods. BMJ. 2024;385:e078378.
(PDF)

S1 Fig Word cloud of final predictors. Visual representation of the frequency of input variables (features) reported across the included studies. The font size of each term is proportional to the number of times it was cited as a final predictor in the models. The prominence of “Image pixels” highlights the dominance of computer vision approaches, followed by sociodemographic (e.g., Age, Gender) and clinical variables.
(PDF)

S2 Fig Landscape of Artificial Intelligence Algorithms. (a) Top 10 most frequently tested AI algorithms during the experimental phase. (b) Top 10 final model architectures selected for the proposed solutions. The bars represent the absolute number of studies utilizing each technique.
(PDF)

S3 Fig Reproducibility: Code and data availability. Assessment of the availability of source code and datasets across the included studies. The categories reflect whether the assets were publicly available, available upon request, or not reported.
(PDF)

S4 Fig Applicability concerns assessment by PROBAST+AI. (a) Distribution of applicability concerns for the model development phase across domains. (b) Distribution of applicability concerns for the model evaluation phase across domains. Green bars indicate low concern, indicating high alignment with the clinical research question.
(PDF)

S1 Appendix Included studies. (PDF)

S2 Appendix Excluded studies at full-text screening. (PDF)

S1 Prisma Checklist PRISMA 2020 checklist for systematic reviews. This checklist is licensed under a Creative Commons Attribution 4.0 International License (CC BY 4.0). Sourced from: Page MJ, McKenzie JE, Bossuyt PM, Boutron I, Hoffmann TC, Mulrow CD, et al. The PRISMA 2020 statement: an updated guideline for reporting systematic reviews. BMJ. 2021;372:n71. https://doi.org/10.1136/bmj.n71. Available at https://www.prisma-statement.org/.
(PDF)
