# Supplementary material for: Artificial intelligence for the diagnosis and prognosis of leprosy, leishmaniasis, and schistosomiasis: A systematic review
Source: PLoS Negl Trop Dis. 2026 Nov 5;20(11):e0014833. doi: 10.1371/journal.pntd.0014833 (PMC13754222; doi:10.1371/journal.pntd.0014833)
Supplement: Supplementary file 2 [file pntd.0014833.s002.pdf]

S2 Table. Full Search Strategy for Each Database

Database	Records Identified	Full Search Query	Search Fields
PubMed (MEDLINE)	202	(("Machine Learning"[MeSH Terms] OR "Artificial Intelligence"[MeSH Terms] OR "Deep Learning"[MeSH Terms] OR "Diagnosis, Computer-Assisted"[MeSH Terms]) OR (machine learning[Title/Abstract] OR artificial intelligence[Title/Abstract] OR deep learning[Title/Abstract] OR prediction model[Title/Abstract] OR predictive model[Title/Abstract] OR transfer learning[Title/Abstract] OR computer-aided diagnosis[Title/Abstract] OR computer-assisted diagnosis[Title/Abstract] OR algorithm*[Title/Abstract])) AND (("Leprosy"[MeSH Terms] OR "Leishmaniasis"[MeSH Terms] OR "Schistosomiasis"[MeSH Terms] OR "Neglected Diseases"[MeSH Terms] OR "Mycobacterium leprae"[MeSH Terms] OR "Leishmania"[MeSH Terms] OR "Schistosoma"[MeSH Terms]) OR (leprosy[Title/Abstract] OR leprae[Title/Abstract] OR Hansen's Disease[Title/Abstract] OR leishmani*[Title/Abstract] OR schistosom*[Title/Abstract] OR bilharzia[Title/Abstract] OR neglected tropical disease[Title/Abstract] OR neglected disease[Title/Abstract]))	MeSH Terms / Title / Abstract
BVS (LILACS, SciELO)	361	("machine learning" OR "artificial intelligence" OR "deep learning" OR "prediction model" OR "predictive model" OR "transfer learning" OR "computer-aided diagnosis" OR "computer-assisted diagnosis" OR algorithm OR algorithms OR "aprendizado de máquina" OR "aprendizagem de máquina" OR "inteligência artificial" OR "aprendizado profundo" OR "modelo preditivo" OR "modelo de predição" OR "diagnóstico auxiliado por computador" OR algoritmo OR algoritmos) AND (leprosy OR leprae OR "Hansen's Disease" OR leishmania OR leishmaniasis OR schistosoma OR schistosomiasis OR bilharzia OR "neglected tropical disease" OR "neglected disease" OR hanseníase OR "doença de Hansen" OR lepra OR leishmania OR leishmaniose OR esquistossomose OR bilharziose OR "doença tropical negligenciada" OR "doença negligenciada")	Title / Abstract
ACM Digital Library	82	query: { AllField:("machine learning" OR "artificial intelligence" OR "deep learning" OR "prediction model" OR "predictive model" OR "transfer learning" OR "computer-aided diagnosis" OR "computer-assisted diagnosis" OR algorithm) AND AllField:(leprosy OR leprae OR "Hansen's Disease" OR leishmania OR leishmaniasis OR schistosoma OR schistosomiasis OR bilharzia OR "neglected tropical disease" OR "neglected disease") }	All fields
IEEE Xplore	53	(("All Metadata":"machine learning" OR "All Metadata":"artificial intelligence" OR "All Metadata":"deep learning" OR "All Metadata":"prediction model" OR "All Metadata":"predictive model" OR "All Metadata":"transfer learning" OR "All Metadata":"computer-aided diagnosis" OR "All Metadata":" computer-assisted diagnosis" OR "All Metadata":"algorithm*")) AND (("All Metadata":leprosy OR "All Metadata":leprae OR "All Metadata":"Hansen's Disease" OR "All Metadata":leishmani* OR "All Metadata": schistosom* OR "All Metadata":bilharzia OR "All Metadata":"neglected tropical disease" OR "All Metadata":"neglected disease"))	All metadata
Web of Science	438	("machine learning" OR "artificial intelligence" OR "deep learning" OR "prediction model" OR "predictive model" OR "transfer learning" OR "computer-aided diagnosis" OR "computer-assisted diagnosis" OR algorithm*) AND (leprosy OR leprae OR "Hansen's Disease" OR leishmani* OR schistosom* OR bilharzia OR "neglected tropical disease" OR "neglected disease")	Keyword / Title / Abstract
**Total Identified**	**1136**		

Abbreviations: ACM, Association for Computing Machinery; BVS, Biblioteca Virtual em Saúde (Virtual Health Library); IEEE, Institute of Electrical and Electronics Engineers; LILACS, Latin American and Caribbean Health Sciences Literature; MeSH, Medical Subject Headings; SciELO, Scientific Electronic Library Online.
