# Supplementary material for: Artificial intelligence for the diagnosis and prognosis of leprosy, leishmaniasis, and schistosomiasis: A systematic review
Source: PLoS Negl Trop Dis. 2026 Nov 5;20(11):e0014833. doi: 10.1371/journal.pntd.0014833 (PMC13754222; doi:10.1371/journal.pntd.0014833)
Supplement: Supplementary file 3 [file pntd.0014833.s003.pdf]

S3 Table. Data Extraction Framework Based on CHARMS Domains

CHARMS Domain	Extracted Variables
**Source of data**	Study Country, Study Design, Data Source, Data Scope, Data Origin
**Participants**	Target Disease, Unit of Analysis
**Outcome(s) to be predicted**	Prediction Task Type, Outcome Subcategory
**Candidate predictors**	Feature Selection Method, Predictor Categories, Final Predictors, Continuous Predictor Handling, Categorical Predictor Handling
**Sample size**	Sample Size
**Missing data**	Missing Data Handling
**Model development**	Imbalance Handling, ML Algorithms Tested, Model Training Approach, Hyperparameter Optimization, Data Split Strategy, Transfer Learning
**Model performance**	AUC-ROC, F1-Score, Specificity, Sensitivity, Precision, Accuracy
**Model evaluation**	External Validation, Baseline Type
**Results**	Final Model
**Interpretation and discussion**	Clinical Utility Assessment, XAI Category, Specific XAI Method
**Additional**	Study ID, Journal, Publication Year, Impact Factor, Funding Source, Analyze Images, Code Availability and Link, Data Availability and Link

Abbreviations: AUC-ROC, Area Under the Receiver Operating Characteristic curve; CHARMS, Checklist for Critical Appraisal and Data Extraction for Systematic Reviews of Prediction Modelling Studies; ID, Identifier; LOOCV, Leave-One-Out Cross-Validation; ML, Machine Learning; XAI, Explainable Artificial Intelligence.
