# Supplementary material for: Artificial intelligence for the diagnosis and prognosis of leprosy, leishmaniasis, and schistosomiasis: A systematic review
Source: PLoS Negl Trop Dis. 2026 Nov 5;20(11):e0014833. doi: 10.1371/journal.pntd.0014833 (PMC13754222; doi:10.1371/journal.pntd.0014833)
Supplement: Supplementary file 4 [file pntd.0014833.s004.pdf]

S4 Table. Data Dictionary

Column Name	Description	Filling
**Study ID**	Unique study identifier.	2-digit sequential number.
**Journal**	Name of the scientific journal.	Full name.
**Publication Year**	Year of publication.	YYYY format.
**Impact Factor**	Journal impact factor.	Numeric value. Journal Citation Reports (JCR) of the year of publication.
**Funding Source**	Main source of funding.	Public (governmental); Industry (corporate); Philanthropic (non-profit); Mixed (multiple sources); No funding; Not reported.
**Study Country**	Country of data origin.	List of countries. If $\geq$4 countries → Multinational. If not reported → Not reported.
**Target Disease**	Target disease(s).	Leprosy; Leishmaniasis; Schistosomiasis.
**Study Design**	Epidemiological design.	Prospective cohort (forward-in-time); Retrospective cohort (backward-in-time); Case-control (outcome-based sampling); Cross-sectional (single time point); Secondary database (uses existing data); Not reported.
**Prediction Task Type**	Task type.	Diagnosis (predicts current condition); Prognosis (predicts future event).
**Outcome Subcategory**	Outcome subtype.	Disease Identification (presence vs. absence); Disease Stratification (subtype/severity); Laboratory Confirmation (pathogen/marker detection); Morbidity and Complications (predicts sequelae); Mortality (predicts death); Unfavorable Clinical Course (relapse/treatment failure).
**Data Source**	Data source.	Primary Research Data (collected for the study); Health Records; Surveillance Databases (public health data); Public Data (open online datasets); Not Reported.
**Data Scope**	Data Scope.	Single-center (one institution); Multi-center (multiple institutions); Not reported.
**Data Origin**	Data origin.	Proprietary (first-party data collected by authors); External (third-party existing data); Mixed (combination of both); Not reported.
**Sample Size**	Total sample size.	Numeric value.
**Unit of Analysis**	Unit of analysis.	Patient; Macroscopic image; Microscopic image; Other.
**Feature Selection Method**	Predictor selection method.	Filter (statistical properties); Wrapper (uses model performance); Embedded (intrinsic to training); Knowledge-based (expert-driven); None; Not applicable.
**Predictor Categories**	Variable categories.	Clinical; Demographic; Epidemiological; Genomic; Image; Laboratory; Other.
**Final Predictors**	Final variables.	List of final model predictors. If there are more than ten → summarized by type. For images → Enter image pixels.
**Continuous Predictor Handling**	Handling of continuous variables.	Transformation (scaling/normalizing); Categorization (binning); None (Kept Continuous); Not Applicable; Not Reported.
**Categorical Predictor Handling**	Handling of categorical variables.	Grouping (combining levels); Dummy / One-Hot Encoding (binary flags); None (Native to Model); Other; Not Applicable; Not Reported.
**Missing Data Handling**	Handling of missing data.	Exclusion - cases (removes rows); Exclusion - features (removes columns); Imputation (fills in values); None (complete data); Not Reported.
**Imbalance Handling**	Handling of imbalance.	Data-level balancing (resampling/augmentation); Algorithm-level balancing (cost-sensitive learning); Balanced by design (e.g., case-control); None (balanced data); Not Reported.
**ML Algorithms Tested**	Algorithms tested.	Algorithms tested.
**Model Training Approach**	Training approach.	Training from Scratch; Transfer Learning (pre-trained weights); Not Applicable.
**Hyperparameter Optimization**	Hyperparameter tuning.	Automated Search (grid/random search); Manual Tuning (trial-and-error); Defined Parameters (no search described); Default Parameters; Not Applicable; Not Reported.
**Data Split Strategy**	Splitting strategy.	k-fold (Cross-validation); LOOCV (Cross-validation); Train/Validation/Test (Hold-out); Train/Test (Hold-out); Other; Not Applicable; Not Reported.
**External Validation**	External validation performed.	Yes; No.
**Analyze Images**	Image data used.	Yes; No.
**Transfer Learning**	Use of transfer learning.	Yes; No.
**Final Model**	Final algorithm or best performance.	Standardized algorithm name. If not reported → Not reported.
**AUC-ROC**	AUC-ROC of the final model.	Numeric value.
**F1-Score**	F1-Score of the final model.	Numeric value.
**Specificity**	Specificity of the final model.	Numeric value.
**Sensitivity**	Sensitivity of the final model.	Numeric value.
**Precision**	Precision of the final model.	Numeric value.
**Accuracy**	Accuracy of the final model.	Numeric value.
**Baseline Type**	Comparator type.	Clinical Standard or Expert (human/existing test); Reference Model (comparison with previous ML/statistical model); No Direct Comparison; Not Reported.
**Clinical Utility Assessment**	Clinical utility assessment.	Clinical Impact Assessment (decision analysis); Clinical Tool Development (app/calculator); Implementation Proof-of-Concept (prototype); Interpretability Analysis (XAI for trust); None (qualitative discussion); Not Reported.
**XAI Category**	Interpretability (XAI) approach.	Interpretable Model; Embedded Feature Importance; Post-hoc Explanation; None; Not Applicable.
**Specific XAI Method**	Specific XAI technique.	Name of XAI technique.(e.g., SHAP; Grad-CAM).
**Code Availability**	Code availability.	Public (open access); Upon request (contact author); Not reported.
**Code Link**	Code link.	URL, only if publicly available.
**Data Availability**	Data availability.	Public (open access); Upon request (contact author); Not reported.
**Data Link**	Data link.	URL, only if publicly available.

Abbreviations: AUC-ROC, Area Under the Receiver Operating Characteristic curve; ID, Identifier; LOOCV, Leave-One-Out Cross-Validation; ML, Machine Learning; XAI, Explainable Artificial Intelligence.
