# Supplementary material for: Artificial intelligence for the diagnosis and prognosis of leprosy, leishmaniasis, and schistosomiasis: A systematic review
Source: PLoS Negl Trop Dis. 2026 Nov 5;20(11):e0014833. doi: 10.1371/journal.pntd.0014833 (PMC13754222; doi:10.1371/journal.pntd.0014833)
Supplement: Supplementary file 5 [file pntd.0014833.s005.pdf]

S5 Table. Reasons for Exclusion of Studies After Full-Text Screening

Reason for Exclusion	Excluded Articles [Reference]	Articles
Ineligible Language	Guo et al. (2024) [4]<br>Zhu et al. (2024) [8]	02
Ineligible Population	Ashour, Amira S et al. (2018) [1]<br>Ashour, Dalia S. et al. (2018) [2]	02
Ineligible Study Scope	Jewani et al. (2018) [5]<br>Rafay & Hussain (2023) [6]	02
Full Text Not Available	Cabunilas et al. (2023) [3]<br>Vargas-Clavijo et al. (2025) [7]	02
**Total Excluded Articles**		**08**

References regarding the excluded studies are presented in S2 Appendix.
