# Supplementary material for: Artificial intelligence for the diagnosis and prognosis of leprosy, leishmaniasis, and schistosomiasis: A systematic review
Source: PLoS Negl Trop Dis. 2026 Nov 5;20(11):e0014833. doi: 10.1371/journal.pntd.0014833 (PMC13754222; doi:10.1371/journal.pntd.0014833)
Supplement: Supplementary file 6 [file pntd.0014833.s006.pdf]

S6 Table. General Characteristics

Characteristic	Category / Measure	Value / Frequency	Percentage (%)
**Journal**	PLOS Neglected Tropical Diseases	8	11.6
	Scientific Reports	4	5.8
	Diagnostics	2	2.9
	BMC Infectious Diseases	2	2.9
	SN Computer Science	2	2.9
	Others	51	73.9
**Publication Year**	2016 - 2019	7	10.1
	2020 - 2025	62	89.9
**Impact Factor**	Mean (SD)	4.47 (3.00)	
	Median (IQR)	3.75 (3.2 –4.41)	
	Range (Min–Max)	1.20–15.70	
	Not available	26	37.7
**Funding Source**	Public	37	53.6
	Not reported	14	20.3
	No funding	10	14.5
	Mixed	5	7.2
	Philanthropic	2	2.9
	Industry	1	1.4
**Study Country**	Brazil	15	21.7
	India	12	17.4
	China	8	11.6
	Colombia	6	8.7
	Others	28	40.6
**Target Disease**	Leprosy	27	39.1
	Schistosomiasis	22	31.9
	Leishmaniasis	17	24.6
	Leishmaniasis; Leprosy	3	4.3
**Study Design**	Secondary database	32	46.4
	Cross-sectional	23	33.3
	Prospective cohort	9	13.0
	Retrospective cohort	4	5.8
	Case-control	1	1.4
**Prediction Task Type**	Diagnosis	56	81.2
	Prognosis	13	18.8
**Outcome Subcategory**	Disease Identification	30	43.5
	Laboratory Confirmation	22	31.9
	Unfavorable Clinical Course	6	8.7
	Disease Stratification	6	8.7
	Morbidity and Complications	3	4.3
	Mortality	2	2.9

Abbreviations: SD, Standard Deviation; IQR, Interquartile Range; Min, Minimum; Max, Maximum.
Note: The category "Diagnosis" in this table corresponds to "Diagnostic Aid" as defined in the methodology, encompassing disease detection and species identification. The category "Prognosis" refers to "Prognosis of Adverse Outcomes", including the prediction of disease progression, complications, and mortality. Disease staging was classified according to its role in predicting clinical evolution versus current status confirmation.
