# Supplementary material for: Artificial intelligence for the diagnosis and prognosis of leprosy, leishmaniasis, and schistosomiasis: A systematic review
Source: PLoS Negl Trop Dis. 2026 Nov 5;20(11):e0014833. doi: 10.1371/journal.pntd.0014833 (PMC13754222; doi:10.1371/journal.pntd.0014833)
Supplement: Supplementary file 7 [file pntd.0014833.s007.pdf]

S7 Table. Data Characteristics

Characteristic	Category / Measure	Value / Frequency	Percentage (%)
**Data Source**	Primary Research Data	36	52.2
	Health Records	19	27.5
	Public Data	10	14.5
	Surveillance Databases	3	4.3
	Not Reported	1	1.4
**Data Scope**	Single-center	35	50.7
	Multi-center	22	31.9
	Not Reported	12	17.4
**Data Origin**	Proprietary	38	55.1
	External	28	40.6
	Mixed	2	2.9
	Not Reported	1	1.4
**Unit of Analysis**	Patient	26	37.7
	Macroscopic image	22	31.9
	Microscopic image	21	30.4
**Sample Size**	Mean (SD)	5542.54 (19578.94)	
	Median (IQR)	515 (172–1524)	
	Range (Min–Max)	34–128,069	
	Not available	4	5.8
**Predictor Categories***	Image	46	66.7
	Clinical	19	27.5
	Laboratory	16	23.2
	Demographic	15	21.7
	Epidemiological	9	13.0
	Genomic	2	2.9

* The sum of frequencies and percentages may exceed the total number of studies (69) and 100%, as a single study may contain multiple values for the same characteristic. The values reflect the count of each individual mention.
Abbreviations: SD, Standard Deviation; IQR, Interquartile Range; Min, Minimum; Max, Maximum.
