# Supplementary material for: Artificial intelligence for the diagnosis and prognosis of leprosy, leishmaniasis, and schistosomiasis: A systematic review
Source: PLoS Negl Trop Dis. 2026 Nov 5;20(11):e0014833. doi: 10.1371/journal.pntd.0014833 (PMC13754222; doi:10.1371/journal.pntd.0014833)
Supplement: Supplementary file 8 [file pntd.0014833.s008.pdf]

S8 Table. Data Pre-processing Characteristics

Characteristic	Category	Frequency	Percentage (%)
**Feature Selection Method***	Not Applicable	34	49.3
	Filter	15	21.7
	Embedded	11	15.9
	None	8	11.6
	Wrapper	7	10.1
	Knowledge-based	4	5.8
**Continuous Predictor Handling**	Not Applicable	35	50.7
	Transformation	13	18.8
	Not Reported	8	11.6
	None (Kept Continuous)	7	10.1
	Categorization	6	8.7
**Categorical Predictor Handling**	Not Applicable	48	69.6
	Not Reported	11	15.9
	None (Native to Model)	7	10.1
	Dummy / One-Hot Encoding	3	4.3
**Missing Data Handling***	Not Reported	46	66.7
	None (complete data)	10	14.5
	Exclusion - cases	9	13.0
	Imputation	3	4.3
	Exclusion - features	3	4.3
**Imbalance Handling**	Not Reported	35	50.7
	Data-level balancing	20	29.0
	Balanced by design	9	13.0
	None (balanced data)	3	4.3
	Algorithm-level balancing	2	2.9
**Data Split Strategy**	Train/Test (Hold-out)	29	42.0
	Train/Validation/Test (Hold-out)	16	23.2
	k-fold (Cross-validation)	12	17.4
	Not Reported	6	8.7
	LOOCV (Cross-validation)	3	4.3
	Not Applicable	3	4.3

* The sum of frequencies and percentages may exceed the total number of studies (69) and 100%, as a single study may contain multiple values for the same characteristic. The values reflect the count of each individual mention.
Abbreviation: LOOCV, Leave-One-Out Cross-Validation.
