# Supplementary material for: Artificial intelligence for the diagnosis and prognosis of leprosy, leishmaniasis, and schistosomiasis: A systematic review
Source: PLoS Negl Trop Dis. 2026 Nov 5;20(11):e0014833. doi: 10.1371/journal.pntd.0014833 (PMC13754222; doi:10.1371/journal.pntd.0014833)
Supplement: Supplementary file 9 [file pntd.0014833.s009.pdf]

S9 Table. Machine Learning Model Details

| Characteristic | Category | Frequency | Percentage (%) |
|---|---|---|---|
| **ML Algorithms Tested*** | Random Forest | 15 | 21.7 |
| | Support Vector Machine | 11 | 15.9 |
| | Logistic Regression | 10 | 14.5 |
| | XGBoost | 9 | 13.0 |
| | Others | 178 | 254.4 |
| **Final Model*** | Logistic Regression | 5 | 7.2 |
| | Artificial Neural Network | 5 | 7.2 |
| | Support Vector Machine | 5 | 7.2 |
| | Random Forest | 4 | 5.8 |
| | Decision Tree | 4 | 5.8 |
| | YOLOv5 | 4 | 5.8 |
| | Others | 48 | 68.1 |
| **Model Training Approach** | Training from Scratch | 43 | 62.3 |
| | Transfer Learning | 21 | 30.4 |
| | Training from Scratch; Transfer Learning | 5 | 7.2 |
| **Hyperparameter Optimization** | Defined Parameters | 26 | 37.7 |
| | Manual Tuning | 16 | 23.2 |
| | Not Reported | 11 | 15.9 |
| | Automated Search | 11 | 15.9 |
| | Not Applicable | 3 | 4.3 |
| | Default Parameters | 2 | 2.9 |
| **External Validation** | No | 57 | 82.6 |
| | Yes | 12 | 17.4 |

* The sum of frequencies and percentages may exceed the total number of studies (69) and 100%, as a single study may contain multiple values for the same characteristic. The values reflect the count of each individual mention.
*Abbreviations:* ML, Machine Learning; XGBoost, eXtreme Gradient Boosting.
