# Supplementary material for: Artificial intelligence for the diagnosis and prognosis of leprosy, leishmaniasis, and schistosomiasis: A systematic review
Source: PLoS Negl Trop Dis. 2026 Nov 5;20(11):e0014833. doi: 10.1371/journal.pntd.0014833 (PMC13754222; doi:10.1371/journal.pntd.0014833)
Supplement: Supplementary file 10 [file pntd.0014833.s010.pdf]

S10 Table. **Study Characteristics and Methodological Indicators Stratified by Target Disease**

Characteristic	Leprosy	Leishmaniasis	Schistosomiasis	Leishmaniasis and Leprosy	Overall
**Total Studies, n (%)**	27 (39.1%)	17 (24.6%)	22 (31.9%)	3 (4.3%)	69 (100.0%)
**Prediction Task**					
Diagnostic Aid	23 (85.2%)	13 (76.5%)	17 (77.3%)	3 (100.0%)	56 (81.2%)
Prognosis	4 (14.8%)	4 (23.5%)	5 (22.7%)	0 (0.0%)	13 (18.8%)
**Data Modality**					
Image-based	16 (59.3%)	14 (82.4%)	12 (54.5%)	3 (100.0%)	45 (65.2%)
Tabular / Clinical	11 (40.7%)	3 (17.6%)	10 (45.5%)	0 (0.0%)	24 (34.8%)
**Model Architecture**					
Deep Learning	9 (33.3%)	10 (58.8%)	11 (50.0%)	1 (33.3%)	31 (44.9%)
Classical & Ensemble ML	15 (55.6%)	4 (23.5%)	8 (36.4%)	1 (33.3%)	28 (40.6%)
Other Models	3 (11.1%)	3 (17.6%)	3 (13.6%)	1 (33.3%)	10 (14.5%)
**Sample Size**					
Median [IQR]	264 [151–876]	172 [126–1036]	1250 [489–4136]	1054 [962–1054]	515 [172–1524]
**Validation Strategy**					
External Validation	4 (14.8%)	1 (5.9%)	7 (31.8%)	0 (0.0%)	12 (17.4%)
**Open Science Practices**					
Source Code Available	5 (18.5%)	6 (35.3%)	3 (13.6%)	2 (66.7%)	16 (23.2%)
Data Available	14 (51.9%)	12 (70.6%)	12 (54.5%)	2 (66.7%)	40 (58.0%)

Note: IQR, Interquartile Range; ML, Machine Learning. Data and source code availability include resources designated as publicly accessible or available upon request.
