# Supplementary material for: Artificial intelligence for the diagnosis and prognosis of leprosy, leishmaniasis, and schistosomiasis: A systematic review
Source: PLoS Negl Trop Dis. 2026 Nov 5;20(11):e0014833. doi: 10.1371/journal.pntd.0014833 (PMC13754222; doi:10.1371/journal.pntd.0014833)
Supplement: Supplementary file 11 [file pntd.0014833.s011.pdf]

S11 Table. Reported Model Performance

Metric	Mean (SD)	Median (IQR)	Range (Min–Max)	Not available, n (%)
**AUC ROC**	0.86 (0.09)	0.86 (0.80–0.94)	0.71–1.00	45 (65.2)
**F1 Score**	0.90 (0.10)	0.93 (0.84–0.98)	0.61–1.00	41 (59.4)
**Specificity**	0.86 (0.14)	0.89 (0.75–0.97)	0.35–1.00	38 (55.1)
**Sensitivity**	0.87 (0.12)	0.90 (0.82–0.97)	0.51–1.00	18 (26.1)
**Precision**	0.88 (0.17)	0.94 (0.83–0.98)	0.19–1.00	38 (55.1)
**Accuracy**	0.90 (0.08)	0.92 (0.86–0.97)	0.71–1.00	25 (36.2)

Abbreviations: SD, Standard Deviation; IQR, Interquartile Range; AUC ROC, Area Under the Receiver Operating Characteristic Curve; Min, Minimum; Max, Maximum.
Note: The mean and median values reported reflect only the subset of studies that disclosed each specific metric. The high percentage of "Not available" data indicates a significant reporting bias in the literature.
