# Supplementary material for: Artificial intelligence for the diagnosis and prognosis of leprosy, leishmaniasis, and schistosomiasis: A systematic review
Source: PLoS Negl Trop Dis. 2026 Nov 5;20(11):e0014833. doi: 10.1371/journal.pntd.0014833 (PMC13754222; doi:10.1371/journal.pntd.0014833)
Supplement: Supplementary file 12 [file pntd.0014833.s012.pdf]

S12 Table. Transparency, Interpretation, and Clinical Utility

Characteristic	Category	Frequency	Percentage (%)
**Baseline Type**	Reference Model	48	69.6
	No Direct Comparison	16	23.2
	Clinical Standard or Expert	5	7.2
**Clinical Utility Assessment***	Implementation Proof-of-Concept	23	33.3
	None (qualitative discussion)	20	29.0
	Clinical Tool Development	19	27.5
	Interpretability Analysis	19	27.5
**XAI Category***	None	36	52.2
	Interpretable Model	16	23.2
	Post-hoc Explanation	14	20.3
	Embedded Feature Importance	5	7.2
**Specific XAI Method***	Not Applicable	36	52.2
	Decision Tree	7	10.1
	Grad-CAM	5	7.2
	Logistic Regression	5	7.2
	SHAP	3	4.3
	Others	19	27.5
**Code Availability**	Not Reported	53	76.8
	Public - URL Available	12	17.4
	Upon Request	4	5.8
**Data Availability**	Not Reported	29	42.0
	Public - URL Available	25	36.2
	Upon Request	15	21.7

* The sum of frequencies and percentages may exceed the total number of studies (69) and 100%, as a single study may contain multiple values for the same characteristic. The values reflect the count of each individual mention.
Abbreviations: XAI, Explainable Artificial Intelligence; Grad-CAM, Gradient-weighted Class Activation Mapping; SHAP, SHapley Additive exPlanations; URL, Uniform Resource Locator.
