# Supplementary material for: Artificial intelligence for the diagnosis and prognosis of leprosy, leishmaniasis, and schistosomiasis: A systematic review
Source: PLoS Negl Trop Dis. 2026 Nov 5;20(11):e0014833. doi: 10.1371/journal.pntd.0014833 (PMC13754222; doi:10.1371/journal.pntd.0014833)
Supplement: Supplementary file 14 [file pntd.0014833.s014.pdf]

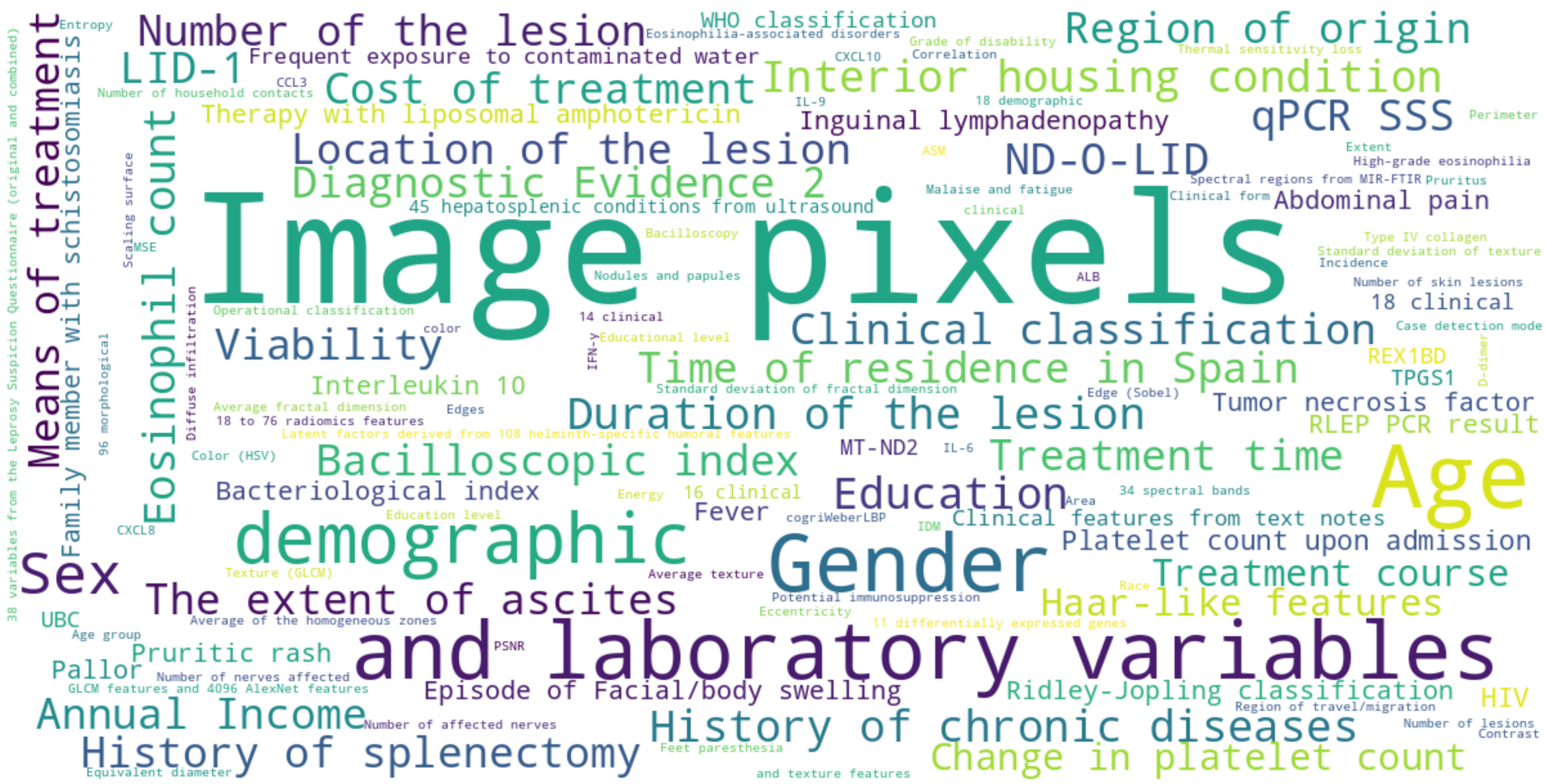

S1 Fig. **Word Cloud of Final Predictors.** Visual representation of the frequency of input variables (features) reported across the included studies. The font size of each term is proportional to the number of times it was cited as a final predictor in the models. The prominence of “Image pixels” highlights the dominance of computer vision approaches, followed by sociodemographic (e.g., Age, Gender) and clinical variables.
