# Supplementary material for: Artificial intelligence for the diagnosis and prognosis of leprosy, leishmaniasis, and schistosomiasis: A systematic review
Source: PLoS Negl Trop Dis. 2026 Nov 5;20(11):e0014833. doi: 10.1371/journal.pntd.0014833 (PMC13754222; doi:10.1371/journal.pntd.0014833)
Supplement: Supplementary file 15 [file pntd.0014833.s015.pdf]

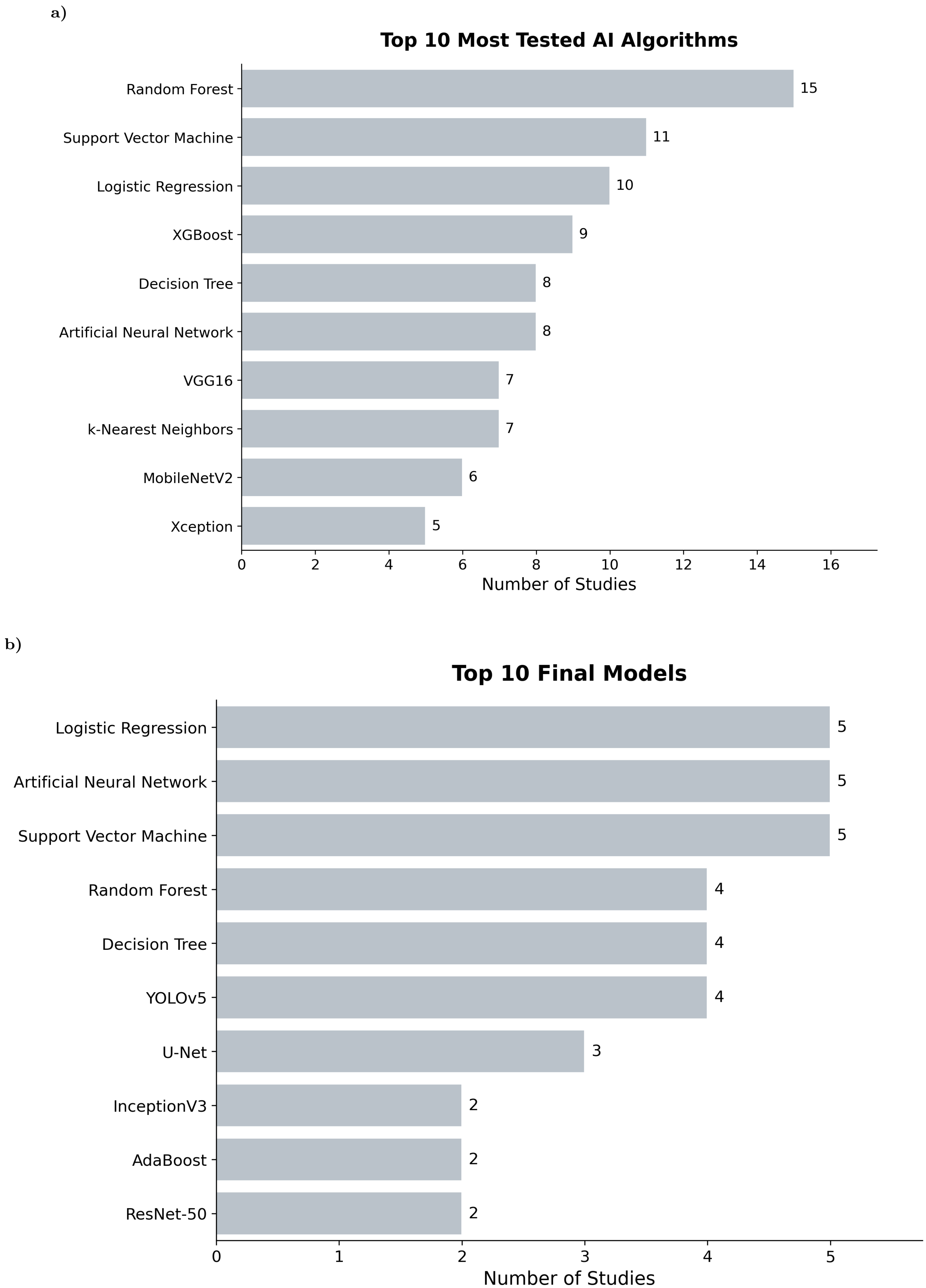

S2 Fig. **Landscape of Artificial Intelligence Algorithms.** (a) Top 10 most frequently tested AI algorithms during the experimental phase. (b) Top 10 final model architectures selected for the proposed solutions. The bars represent the absolute number of studies utilizing each technique.
