# Supplementary material for: Artificial intelligence for the diagnosis and prognosis of leprosy, leishmaniasis, and schistosomiasis: A systematic review
Source: PLoS Negl Trop Dis. 2026 Nov 5;20(11):e0014833. doi: 10.1371/journal.pntd.0014833 (PMC13754222; doi:10.1371/journal.pntd.0014833)
Supplement: Supplementary file 16 [file pntd.0014833.s016.pdf]

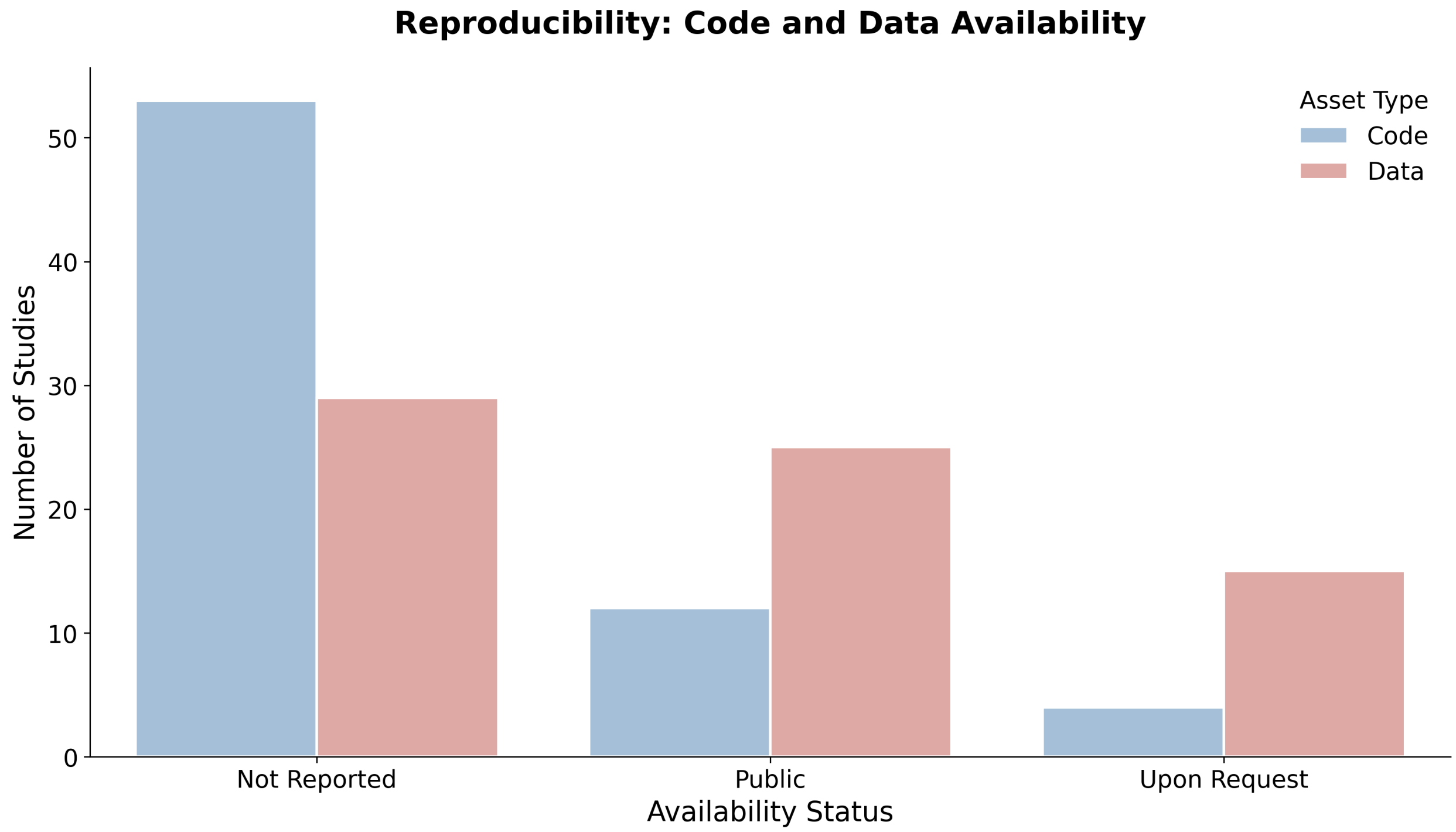

S3 Fig. **Reproducibility: Code and Data Availability.** Assessment of the availability of source code and datasets across the included studies. The categories reflect whether the assets were publicly available, available upon request, or not reported.
