# Supplementary material for: Artificial intelligence for the diagnosis and prognosis of leprosy, leishmaniasis, and schistosomiasis: A systematic review
Source: PLoS Negl Trop Dis. 2026 Nov 5;20(11):e0014833. doi: 10.1371/journal.pntd.0014833 (PMC13754222; doi:10.1371/journal.pntd.0014833)
Supplement: Supplementary file 17 [file pntd.0014833.s017.pdf]

a)

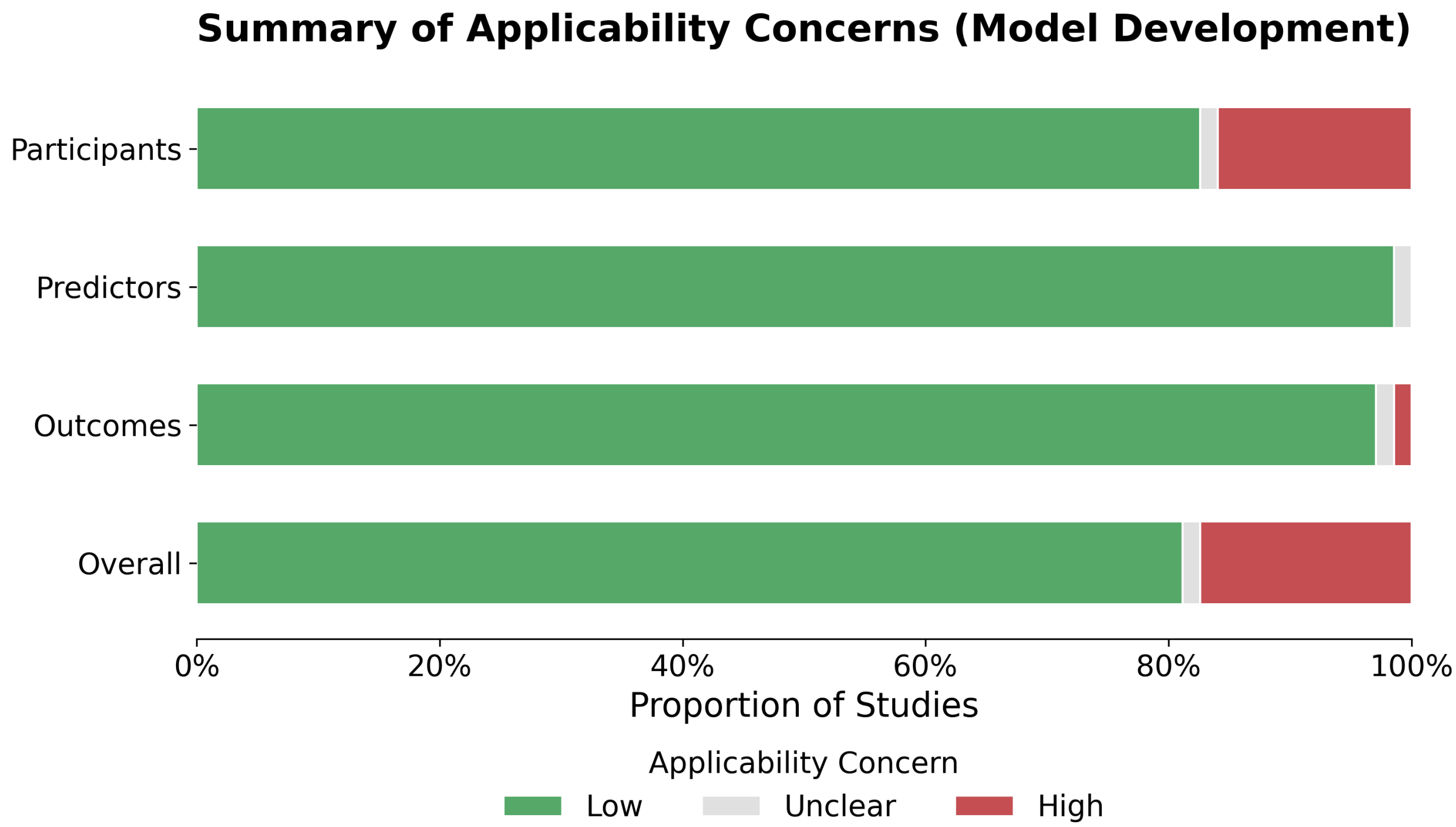

b)

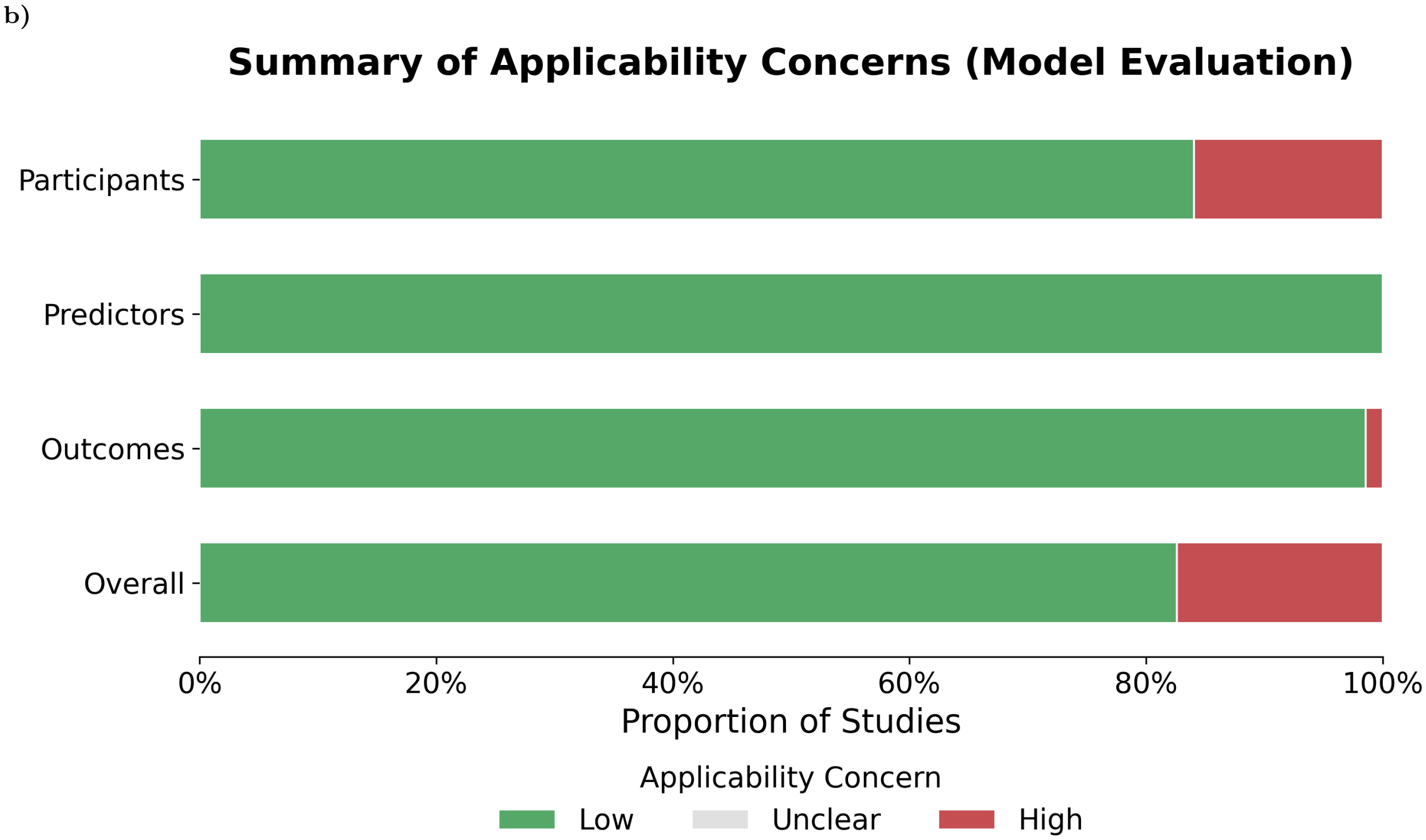

S4 Fig. **Applicability Concerns Assessment by PROBAST+AI.** (a) Distribution of applicability concerns for the model development phase across domains. (b) Distribution of applicability concerns for the model evaluation phase across domains. Green bars indicate low concern, indicating high alignment with the clinical research question.
