# Supplementary material for: Artificial intelligence for the diagnosis and prognosis of leprosy, leishmaniasis, and schistosomiasis: A systematic review
Source: PLoS Negl Trop Dis. 2026 Nov 5;20(11):e0014833. doi: 10.1371/journal.pntd.0014833 (PMC13754222; doi:10.1371/journal.pntd.0014833)
Supplement: Supplementary file 18 [file pntd.0014833.s018.pdf]

S1 APPENDIX - Included Studies

The following list presents the 69 studies that met the eligibility criteria and were included for analysis in this systematic review. The number assigned to each reference corresponds to its unique study identifier (Study ID) in the analysis tables.

1. Sadeghi A, Sadeghi M, Fakhar M, Zakariaei Z, Sadeghi M, Bastani R. A Deep Learning-Based Model for Detecting Leishmania Amastigotes in Microscopic Slides: A New Approach to Telemedicine. BMC Infect Dis 2024; 24: 551.

2. Fernández-Martínez JL, Boga JA, de Andrés-Galiana E, et al. A Machine Learning Model for Evaluating Imported Disease Screening Strategies in Immigrant Populations. Am J Trop Med Hyg 2021; 105: 1413–19.

3. Zare M, Akbarialiabad H, Parsaei H, et al. A Machine Learning-Based System for Detecting Leishmaniasis in Microscopic Images. BMC Infect Dis 2022; 22: 48.

4. Bamorovat M, Sharifi I, Rashedi E, et al. A Novel Diagnostic and Prognostic Approach for Unresponsive Patients with Anthroponotic Cutaneous Leishmaniasis Using Artificial Neural Networks. PloS One 2021; 16: e0250904.

5. Gama RS, de Souza MLM, Sarno EN, et al. A Novel Integrated Molecular and Serological Analysis Method to Predict New Cases of Leprosy amongst Household Contacts. PLoS Negl Trop Dis 2019; 13: e0007400.

6. Ali Z, Hayat MF, Shaukat K, et al. A Proposed Framework for Early Prediction of Schistosomiasis. Diagnostics (Basel) 2022; 12.

7. Ward P, Dahlberg P, Lagatie O, et al. Affordable Artificial Intelligence-Based Digital Pathology for Neglected Tropical Diseases: A Proof-of-Concept for the Detection of Soil-Transmitted Helminths and Schistosoma Mansoni Eggs in Kato-Katz Stool Thick Smears. PLoS Negl Trop Dis 2022; 16: e0010500.

8. Jaikishore CN, Udutalapally V, Das D. AI Driven Edge Device for Screening Skin Lesion and Its Severity in Peripheral Communities. In: 2021 IEEE 18th India Council International Conference (INDICON). 2021: 1–6.

9. Kumar A, Mondal S, Khatua D, et al. AI-driven analysis by identifying risk factors of VL relapse in HIV co-infected patients. Sci Rep 2025; 15.

10. Marçal PHF, de Souza MLM, Gama RS, et al. Algorithm Design for a Cytokine Release Assay of Antigen-Specific In Vitro Stimuli of Circulating Leukocytes to Classify Leprosy Patients and Household Contacts. Open Forum Infect Dis 2022; 9: ofac036.

11. Mduluza-Jokonya TL, Vengesai A, Midzi H, et al. Algorithm for Diagnosis of Early Schistosoma Haematobium Using Prodromal Signs and Symptoms in Pre-School Age Children in an Endemic District in Zimbabwe. PLoS Negl Trop Dis 2021; 15: e0009599.

12. Chaitanya VS, Cuello L, Das M, et al. Analysis of a Novel Multiplex Polymerase Chain Reaction Assay as a Sensitive Tool for the Diagnosis of Indeterminate and Tuberculoid Forms of Leprosy. Int J Mycobacteriol 2017; 6: 1–8.

13. Zhou X, Wang H, Xu C, et al. Application of kNN and SVM to Predict the Prognosis of Advanced Schistosomiasis. Parasitol Res 2022; 121: 2457–60.

14. Pattnayak P, Mohanty A, Das T, Patnaik S. Applying Artificial Intelligence and Deep Learning to Identify Neglected Tropical Skin Disorders. In: 2024 3rd International Conference for Innovation in Technology (INOCON). 2024: 1–6.

15. Abdelmula AM, Mirzaei O, Güler E, Süer K. Assessment of Deep Learning Models for Cutaneous Leishmania Parasite Diagnosis Using Microscopic Images. Diagnostics (Basel) 2023; 14.

16. Monroy B, Sanchez K, Arguello P, et al. Automated Chronic Wounds Medical Assessment and Tracking Framework Based on Deep Learning. Comput Biol Med 2023; 165: 107335.

17. delas Peñas KE, Villacorte EA, Rivera PT, Naval PC. Automated Detection of Helminth Eggs in Stool Samples Using Convolutional Neural Networks. In: 2020 IEEE REGION 10 CONFERENCE (TENCON). 2020: 750–55.

18. Leal JFC, Barroso DH, Trindade NS, de Miranda VL, Gurgel-Gonçalves R. Automated Identification of Cutaneous Leishmaniasis Lesions Using Deep-Learning-Based Artificial Intelligence. Biomedicines 2023; 12.

19. Gonçalves C, Andrade N, Borges A, et al. Automatic detection of Visceral Leishmaniasis in humans using Deep Learning. Signal Image Video Process 2023; 17: 3595–601.

20. Tió-Coma M, Kielbasa SM, van den Eeden SJF, et al. Blood RNA Signature RISK4LEP Predicts Leprosy Years before Clinical Onset. EBioMedicine 2021; 68: 103379.

21. Mehta J, Dharamsey J, et al. Clinical Text Mining of Electronic Health Records to Classify Leprosy Patients Cases. Int J Innov Technol Explor Eng 2020; 9: 2331–36.

22. Zhi YC, Mpooya S, Kabatereine NB, Nabatte B, Opio CK, Chami GF. Clinically validated graphical approaches identify hepatosplenic multimorbidity in individuals at risk of schistosomiasis. R Soc Open Sci 2025; 12.

23. Li G, Zhou X, Liu J, et al. Comparison of Three Data Mining Models for Prediction of Advanced Schistosomiasis Prognosis in the Hubei Province. PLoS Negl Trop Dis 2018; 12: e0006262.

24. Jin B, Cruz L, Gonçalves N. Deep Facial Diagnosis: Deep Transfer Learning From Face Recognition to Facial Diagnosis. IEEE Access 2020; 8: 123649–61.

25. Saha A, Chakraborty T, Rahimikollu J, et al. Deep Humoral Profiling Coupled to Interpretable Machine Learning Unveils Diagnostic Markers and Pathophysiology of Schistosomiasis. Sci Transl Med 2024; 16: eadk7832.

26. Yotsu RR, Ding Z, Hamm J, Blanton RE. Deep Learning for AI-Based Diagnosis of Skin-Related Neglected Tropical Diseases: A Pilot Study. PLoS Negl Trop Dis 2023; 17: e0011230.

27. Oyibo P, Meulah B, Agbana T, et al. Deep learning-based automated detection and multiclass classification of soil-transmitted helminths and Schistosoma mansoni eggs in fecal smear images. Sci Rep 2025; 15.

28. Tekle E, Dese K, Girma S, Adissu W, Krishnamoorthy J, Kwa T. DeepLeish: A Deep Learning Based Support System for the Detection of Leishmaniasis Parasite from Giemsa-Stained Microscope Images. BMC Med Imaging 2024; 24: 152.

29. Chielle EO, Kuiava EL, Faust GA, Chielle APO, Kuiava VA, Alves RJF. Desenvolvimento de sistema estruturado com inteligência artificial para apoio no diagnóstico de parasitoses intestinais. Clin Biomed Res 2020; 40: 148–53.

30. Jaramillo DC, Escobar JE, Galeano J, Torres-Madronero MC. Design of a Multilayer Neural Network for the Classification of Skin Ulcers' Hyperspectral Images: a Proof of Concept. In: Romero E, Romero N, Brieva J, editors. 2020.

31. Mohammed MA, Anzaku ET, Ward PK, et al. Detecting soil-transmitted helminth and Schistosoma mansoni eggs in Kato-Katz stool smear microscopy images: A comprehensive in- and out-of-distribution evaluation of YOLOv7 variants. PLoS Negl Trop Dis 2025; 19.

32. Surasinghe P, Sabapathippillai P, Thanikasalam K. Detection and Visualization of Neglected Tropical Skin Diseases Using EfficientNet and Grad-CAM. In: 2023 5th International Conference on Advancements in Computing (ICAC). 2023: 472–77.

33. Gonçalves C, Borges A, Dias V, et al. Detection of Human Visceral Leishmaniasis Parasites in Microscopy Images from Bone Marrow Parasitological Examination. Appl Sci (Basel) 2023; 13.

34. Agbana TE, Nijman P, Hoeber M, et al. Detection of Schistosoma haematobium using lensless imaging & flow cytometry, a proof of principle study. In: Cote GL, editor. 2020.

35. Simões MR, Lima FR, Lugão HB, et al. Development and Validation of a Machine Learning Approach for Screening New Leprosy Cases Based on the Leprosy Suspicion Questionnaire. Sci Rep 2025; 15: 6912.

36. Novack AC, Cobre AD, Stremel DP, Ferreira LM, Campos ML, Pontarolo R. Development and validation of a new method by MIR-FTIR and chemometrics for the early diagnosis of leprosy and evaluation of the treatment effect. Chemometr Intell Lab Syst 2024; 254.

37. Rubio Maturana C, de Oliveira AD, Zarzuela F, et al. Development of an Automated Artificial Intelligence-Based System for Urogenital Schistosomiasis Diagnosis Using Digital Image Analysis Techniques and a Robotized Microscope. PLoS Negl Trop Dis 2024; 18: e0012614.

38. Contreras-Ramírez M, Sora-Cardenas J, Colorado-Salamanca C, Ovalle-Bracho C, Suárez DR. Enhanced Detection of Leishmania Parasites in Microscopic Images Using Machine Learning Models. Sensors (Basel) 2024; 24.

39. Gawali CJ, Subbulakshmi S. Enhancing Diagnosis of Infectious Skin Disease: A Professional Approach using Deep Learning for Leprosy Detection. In: 2024 15th International Conference on Computing Communication and Networking Technologies (ICCCNT). 2024: 1–7.

40. Kumar Y, Garg P, Moudgil MR, et al. Enhancing Parasitic Organism Detection in Microscopy Images through Deep Learning and Fine-Tuned Optimizer. Sci Rep 2024; 14: 5753.

41. Nyatte S, Perabi S, Abessolo G, Essiane SN, Ele P. Enhancing the Diagnosis of Skin Neglected Tropical Diseases by Artificial Neural Networks Using Evolutionary Algorithms: Implementation on Raspberry Pi. In: Triwiyanto T, Rizal A, Caesarendra W, editors. Proceeding of the 3rd International Conference on Electronics, Biomedical Engineering, and Health Informatics. Springer Nature; 2023.

42. de Freitas LRS, de Freitas JAO, Penna GO, Duarte EC. Evaluating Machine Learning Models for Predicting Late Leprosy Diagnosis by Physical Disability Grade in Brazil (2018-2022). Trop Med Infect Dis 2025; 10.

43. Meulah B, Hoekstra PT, Popoola S, et al. Evaluation of the AiDx Assist Device for Automated Detection of Schistosoma Eggs in Stool and Urine Samples in Nigeria. Front Parasitol 2025; 4: 1440299.

44. Guo Z, Zhao M, Liu Z, et al. Feasibility of Ultrasound Radiomics Based Models for Classification of Liver Fibrosis Due to Schistosoma Japonicum Infection. PLoS Negl Trop Dis 2024; 18: e0012235.

45. Jitendra R, Simha JB, Abhi S, Chadha VK. Heuristic Segmentation Assisted Deep-Spatial Feature Learning Model for Leprosy Detection. SN Comput Sci 2024; 5: 737.

46. Carvalho JC, Pascoal-Xavier MA, Araújo MG, et al. Immune Mediators as Plasma Biomarkers for Identifying Household Contacts and Classifying Clinical Forms and Leprosy Reactions. Front Immunol 2025; 16: 1513060.

47. Mondal B, Das N, Santosh KC, Nasipuri M. Improved Skin Disease Classification Using Generative Adversarial Network. In: 2020 IEEE 33rd International Symposium on Computer-Based Medical Systems (CBMS). 2020: 520–25.

48. Gómez MA, Belew AT, Vargas DA, et al. Innate biosignature of treatment failure in human cutaneous leishmaniasis. Nat Commun 2025; 16.

49. Górriz M, Aparicio A, Raventós B, Vilaplana V, Sayrol E, López-Codina D. Leishmaniasis Parasite Segmentation and Classification Using Deep Learning. In: Perales FJ, Kittler J, editors. Articulated Motion and Deformable Objects. Springer International Publishing; 2018.

50. Baweja AK, Aditya S, Kanchana M. Leprosy Diagnosis using Explainable Artificial Intelligence Techniques. In: 2023 International Conference on Sustainable Computing and Data Communication Systems (ICSCDS). 2023: 551–56.

51. Baweja HS, Parhar T. Leprosy lesion recognition using convolutional neural networks. In: 2016 International Conference on Machine Learning and Cybernetics (ICMLC). 2016; 1: 141–45.

52. De Souza MLM, Lopes GA, Branco AC, Fairley JK, De Oliveira Fraga LA. Leprosy Screening Based on Artificial Intelligence: Development of a Cross-Platform App. JMIR Mhealth Uhealth 2021; 9: e23718.

53. Beesetty R, Reddy SA, Modali S, et al. Leprosy Skin Lesion Detection: An AI Approach Using Few Shot Learning in a Small Clinical Dataset. Indian J Lepr 2023; 95: 89–102.

54. Jiang H, Deng W, Zhou J, et al. Machine Learning Algorithms to Predict the 1 Year Unfavourable Prognosis for Advanced Schistosomiasis. Int J Parasitol 2021; 51: 959–65.

55. Gonçalves CDA, Borges AL, Dias VBL, de Andrade NB, Aguiar BGA, e Silva RRV. Método automático para detecção de Leishmaniose Visceral em humanos. In: XXIV Congresso Brasileiro de Automática. 2022.

56. Nyatte S, Steve P, Ghislain M, Ndjakomo S, Ele P. Optimized real-time diagnosis of neglected tropical diseases by automatic recognition of skin lesions. Informatics in Medicine Unlocked 2022; 33: 101078.

57. Wu X, Ren J, Gao Z, et al. Plasma D-Dimer Can Effectively Predict the Prospective Occurrence of Ascites in Advanced Schistosomiasis Japonica Patients. Korean J Parasitol 2017: 167–74.

58. Mehta J, Kalla M. Predicting Severity from Electronic Health Records of Leprosy Patients using Ensemble Learning. In: 2023 World Conference on Communication & Computing (WCONF). 2023: 1–6.

59. van Waasdijk M, Werff SDV, Sjoholm D, et al. Prediction of helminthiases in travellers and migrants with eosinophilia: a cohort study. Clin Microbiol Infect 2025; 31: 113–20.

60. Arce-Lopera CA, Diaz-Cely J, Quintero L. Presumptive diagnosis of cutaneous leishmaniasis. Front Health Inform 2021; 10: 75.

61. Huo Y, Zhang J, Du X, Wang X, Liu J, Liu L. Recognition of parasite eggs in microscopic medical images based on YOLOv5. In: 2021 5th Asian Conference on Artificial Intelligence Technology (ACAIT). 2021: 123–27.

62. He W, Zhu HY, Geng JJ, et al. Recognition of parasitic helminth eggs via a deep learning-based platform. Front Microbiol 2024; 15.

63. De Goma JC, Devaraj M. Recognizing Common Skin Diseases in the Philippines Using Image Processing and Machine Learning Classification. In: Proceedings of the 2020 3rd International Conference on Computing and Big Data (ICCBD '20). 2020: 68–72.

64. Barbieri RR, Xu YX, Setian L, et al. Reimagining leprosy elimination with AI analysis of a combination of skin lesion images with demographic and clinical data. Lancet Reg Health Am 2022; 9.

65. Pugazhenthi V, Naik SK, Joshi AD, et al. Skin Disease Detection And Classification. Int J Adv Eng Res Sci 2019; 6: 396–400.

66. Banerjee A, Sarkar S, Nasipuri M, Das N. Skin Diseases Detection Using LBP and WLD: An Ensembling Approach. SN Comput Sci 2023; 5: 72.

67. Oliveira BAS, Coelho PRS, Moreira JMP, et al. Towards effective and efficient machine learning models for schistosomiasis diagnosis in microscopic images. Exp Parasitol 2025; 275.

68. Jostan JR, Rodriguez LM, Bernal DZ, et al. Ultrasound Nerve Segmentation with Deep Learning for Leprosy. In: 2025 IEEE 22nd International Symposium on Biomedical Imaging (ISBI). 2025: 1–5.

69. Bamorovat M, Sharifi I, Tahmouresi A, Afshari SAK, Rashedi E. Unlocking Responsive and Unresponsive Signatures: A Transfer Learning Approach for Automated Classification in Cutaneous Leishmaniasis Lesions. Transbound Emerg Dis 2025.
