# Supplementary material for: Artificial intelligence for the diagnosis and prognosis of leprosy, leishmaniasis, and schistosomiasis: A systematic review
Source: PLoS Negl Trop Dis. 2026 Nov 5;20(11):e0014833. doi: 10.1371/journal.pntd.0014833 (PMC13754222; doi:10.1371/journal.pntd.0014833)
Supplement: Supplementary file 19 [file pntd.0014833.s019.pdf]

S2 APPENDIX - Excluded Studies at Full-Text Screening

1. Ashour AS, Hawas AR, Guo Y. Comparative Study of Multiclass Classification Methods on Light Microscopic Images for Hepatic Schistosomiasis Fibrosis Diagnosis. Health Inf Sci Syst 2018; 6: 7.

2. Ashour DS, Abou Rayia DM, Ata MM, Ashour AS, Abd Elnaby MM. Hybrid Feature Extraction Techniques for Microscopic Hepatic Fibrosis Classification. Microscopy Research and Technique 2018; 81: 338–47.

3. Cabunilas JJL, Ponio KJP, Villaverde JF, Dofitas BL, Juanatas RA. Detection of Acid-Fast Bacilli AFB in Slit-Skin Smear Microscopy using MASK R-CNN Algorithm. In: Proceedings of the 2023 13th International Conference on Biomedical Engineering and Technology. 2023: 30–36.

4. Guo Z, Shao J, Zou X, et al. Development of a grading diagnostic model for schistosomiasis-induced liver fibrosis based on radiomics and clinical laboratory indicators. Chinese journal of schistosomiasis control 2024; 36: 251–58.

5. Jewani K, Boddu K, Gumani P, Solapure K. Detection of diseases via blood analysis using Image processing Techniques. In: 2018 International Conference on Smart City and Emerging Technology (ICSCET). 2018: 1–4.

6. Rafay A, Hussain W. EfficientSkinDis: An EfficientNet-based classification model for a large manually curated dataset of 31 skin diseases. Biomedical Signal Processing and Control 2023; 85: 104869.

7. Vargas-Clavijo M, Cardona-Castro N, Ospina-Gómez JP, Serrano-Coll H. Evaluating Advanced Machine Learning Models for Histopathological Diagnosis of Hansen Disease. The American Journal of Dermatopathology 2025; 47: 301–7.

8. Zhu H, Li Y, Zhu D, et al. Establishment and application of an artificial intelligence-assisted platform for detection of parasite eggs. Chinese journal of schistosomiasis control 2024; 36: 643–48.
